# Cohort mortality forecasts indicate signs of deceleration in life expectancy gains

**DOI:** 10.1073/pnas.2519179122

**Published:** 2025-08-25

**Authors:** José Andrade, Carlo Giovanni Camarda, Héctor Pifarré i Arolas

**Affiliations:** ^a^Max Planck Institute for Demographic Research, Rostock 18057, Germany; ^b^Institut national d’Ã©tudes dÃ©mographiques, Aubervilliers 93322, France; ^c^La Follette School of Public Affairs, University of Wisconsin-Madison, Madison, WI 53706; ^d^Center for Demography and Ecology, University of Wisconsin-Madison, Madison, WI 53706; ^e^Center for Demography of Health and Aging, University of Wisconsin-Madison, Madison, WI 53706

**Keywords:** human lifespan, cohort life expectancy, mortality forecasting, slowdown in mortality improvements

## Abstract

The fast-paced improvements in mortality in high-income countries since the early 1900s have led to a sustained increase in life expectancy. However, whether this linear trend will continue or life expectancy gains will decelerate in the near future remains unclear. To answer this question, we apply multiple established and recently developed mortality forecasting methods to estimate cohort life expectancy for individuals born between 1939 and 2000 in 23 high-income countries. Across all forecasting methods, our results robustly and consistently indicate a deceleration in cohort life expectancy. The previously observed pace of improvement, 0.46 y per cohort, declines by 37% to 52%, depending on the method used. Robustness checks suggest that these findings are unlikely to be solely due to downward bias in cohort life expectancy forecasts. Furthermore, an age-decomposition analysis indicates that this deceleration is primarily driven by a slower pace of mortality improvement at very young ages. Over half of the total deceleration is attributable to mortality trends under age 5, while more than two-thirds is explained by mortality trends under age 20. This pattern had already emerged in the observed data for the cohorts included in our analysis. Thus, even if these estimates turned out to be overly pessimistic, it is unlikely that the deceleration will reverse in the near future.

## Significance

Longevity, measured by cohort life expectancy, has risen steadily in high-income countries since the early 1900s, raising expectations that this trend will continue. However, whether these gains will persist remains uncertain. Using multiple mortality forecasting methods, we find robust evidence that currently living cohorts, born between 1939 and 2000, may experience substantially slower gains. This slowdown is driven mainly by a diminished pace of mortality improvements at very young ages, with over half of the deceleration linked to trends under age 5. As this pattern is already evident in observed data, even if our forecasts are overly pessimistic, a reversal is unlikely. These findings have important implications for future longevity projections and social policy.

The limits of human lifespan remain a topic of ongoing debate. Although progress in public health, medical advancements, and socioeconomic development has significantly increased life expectancy in high-income countries over the past century (1, 2), whether this increase will continue along a linear trajectory or slow down remains a matter of active discussion. Several studies have documented that life expectancy has risen steadily and linearly, and argue that this trend is expected to persist into the future (3–7). Others propose that human lifespan is inherently constrained by natural limits, leading to the expectation of a deceleration in life expectancy gains over time (8–11). While most of the literature has examined mortality decline through a period framework (1, 4, 5, 7, 12–14), we study the deceleration hypothesis through a cohort perspective. We employ multiple methods to forecast future mortality for currently living cohorts in high-income, low-mortality countries and use these forecasts to calculate their cohort life expectancy.

The key advantage of cohort life expectancy is that it offers a direct measure of longevity, capturing the actual survival experiences of individuals born in a given year. In contrast, period life expectancy is a synthetic measure that reflects the average mortality risk observed within a specific calendar year (6). This distinction is consequential: A deceleration in period life expectancy does not necessarily signal a slowdown in the pace of longevity gains. For instance, several mortality crises in the early 20th century caused temporary declines in period life expectancy, yet did not lead to a slowdown in cohort life expectancy gains (7). Longer-term trends in mortality reductions more than offset these short-term shocks, resulting in steady gains in longevity for the affected cohorts. Likewise, it remains unclear whether the recent slowdowns (15, 16) and reversals documented in period life expectancy will translate into slower gains in cohort life expectancy. Thus, while period measures may suggest adverse mortality trends, only a cohort perspective can definitively assess longevity.

Despite the inherent advantages of a cohort perspective, studies using cohort measures remain far less common than period-based investigations, with a few notable exceptions (3, 6). The main limitation of observed cohort life expectancy is that it can only be obtained for historical or completed cohorts, which may not reflect the mortality prospects of currently living populations. Most existing work reports cohort life expectancy trends up to the 1938 cohort, with only one study extending the analysis to the 1950 cohort (6). These cohorts experienced decades of rapid mortality decline, despite living through major mortality crises such as the Spanish influenza pandemic and the World Wars. It remains unclear whether the longevity gains observed in these cohorts will extend to more recent ones. Will currently living cohorts achieve similar gains in life expectancy? To address this question, we must complete the remaining mortality schedules of these cohorts. We do so by harnessing multiple established and newly developed forecasting approaches. Our design, like that of Vaupel et al. (4), involves forecasting mortality rates to estimate life expectancy, but differs in adopting a fully cohort-based perspective rather than a period-based one.

We estimate cohort life expectancy for currently living cohorts in 23 high-income, low-mortality countries. To achieve this, we forecast age-specific mortality rates using six distinct methods and subsequently calculate the cohort life expectancies implied by these forecasts, focusing on both the best-practice and median countries. We apply four period-based methods—Lee–Carter (LC) (17), Smooth Constrained Mortality Forecasting CP-Splines (CPS) (18), Compositional Data Analysis (CoDa) (19), and the United Nations World Population Prospects 2024 (WPP) (20)—by extracting the Lexis diagonal to approximate cohort mortality. Additionally, we employ two cohort-specific methods, Linear Lee–Carter (LLC), and Cohort Segmented Transformation Age-at-death Distributions (C-STAD) (21), to directly capture cohort-specific mortality trends. The analysis focuses on cohorts born between 1939 and 2000, using data from the Human Mortality Database (22) (see *Materials and Methods* and *SI Appendix*, Table S5, for details). Finally, we apply decomposition techniques to examine and understand the contributions by different age groups to the forecasted cohort life expectancy trends.

Our forecasts reveal robust and significant deceleration in longevity gains across all methods, in direct contrast with prior findings in the literature based on period approaches (4–7). While the precise magnitude of the deceleration depends on the method, the results are not driven by specific country experiences, are overwhelmingly robust to potential concerns over systematic underestimation, and are not sensitive to forecasting errors up to a factor of 2.

## Results

### Forecasting.

**Fig. 1.**
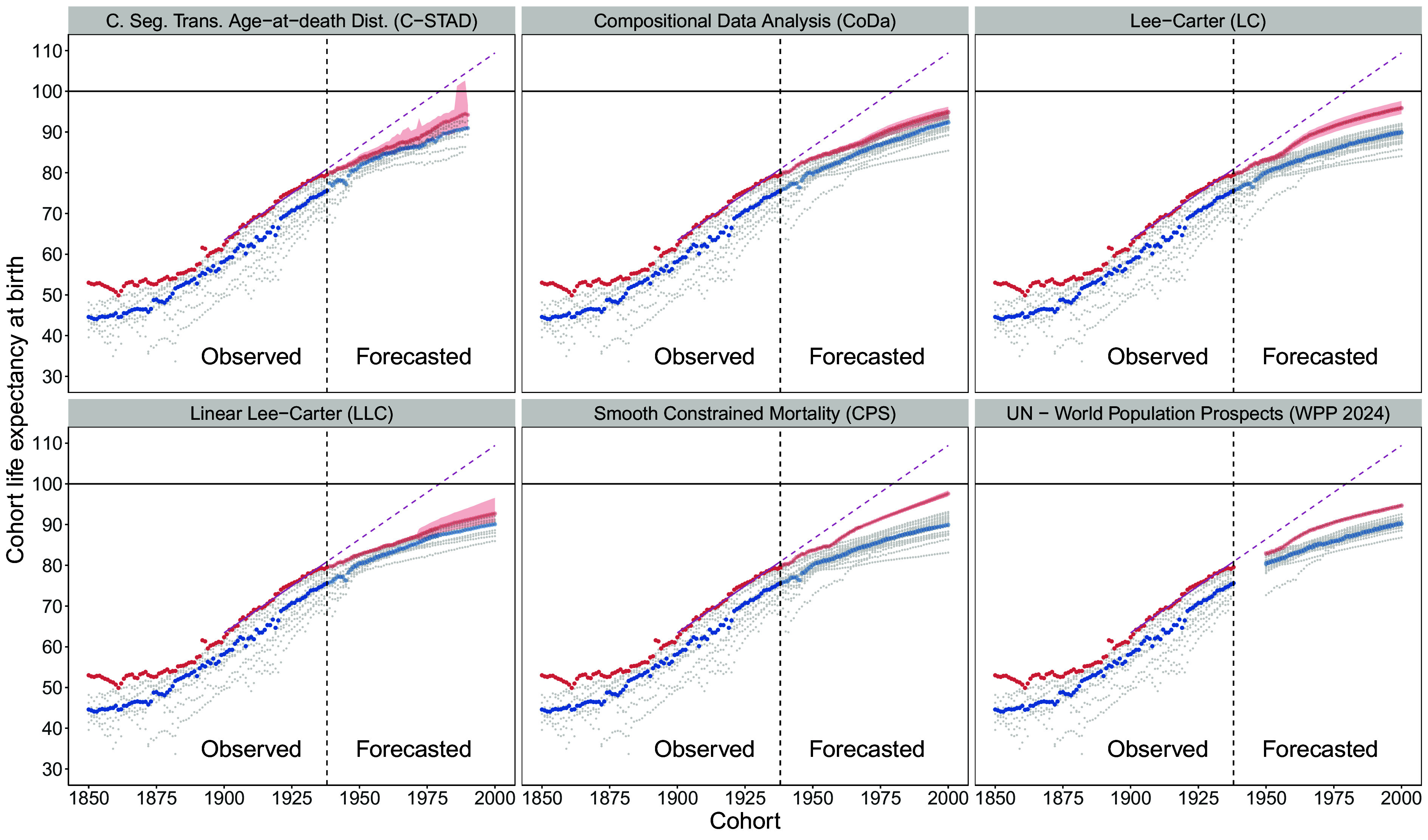
Cohort life expectancy, observed and forecasted. Observed values and forecasted values are separated by the vertical dashed line (black) in 1938. Best-practice (red), median (blue), country-specific (gray), linear extrapolation of best-practice 1900–1938 (pink).

Fig. 1 displays female cohort life expectancy at birth for cohorts born between 1850 and 2000, with each panel showing results for a specific forecasting method. Following previous literature (6), we close life tables at 85+, balancing the inclusion of additional cohorts with a sufficiently high closing age. Given data availability and the open age interval of 85+, the last fully observed cohort was born in 1938 (indicated by the vertical black dashed line). For cohorts born after 1938, life expectancy is estimated through mortality forecasting from their last observed age to 100, and the results are then used to close the life table at age 85 and older (additional details are provided in *Materials and Methods*). Across countries, the best-practice country or highest cohort life expectancy (red), as well as the median (blue), are highlighted (a complete list of best-practice and median countries by cohort and method is provided in *SI Appendix*, Table S4). The median is reported to ensure that our findings are not based on outliers.

For cohorts born after 1938, two predictions are presented. The first prediction, represented by the pink dashed line, involves a linear extrapolation of cohort life expectancy gains observed for cohorts born between 1900 and 1938 for both the best-practice and median cohort life expectancies. This corresponds to the optimistic scenario (3, 5–7), under which cohort life expectancy would continue to increase at a rate of 0.46 y per cohort (the slope of the pink line). Under this scenario, the milestone of a cohort life expectancy of 100 would be achieved as early as the 1980 cohort. For the linear extrapolation of the median, the rate of improvement is higher, at 0.48 y. This is partly driven by the compression of cohort life expectancy across countries during the period.

The second prediction is based on our mortality forecasts using each specific method and includes 95% bootstrapped prediction intervals (PIs, indicated by the shaded area). The pace of improvement in best-practice and median cohort life expectancy in this prediction is determined by the slope of a fitted line to their respective forecasts. By contrast, this prediction indicates a deceleration in the pace of improvement across all methods. For example, the *Bottom*-center panel in Fig. 1 displays the results for the CPS approach. Based on this method, there is a gain of 0.29 y (95% PI: 0.28, 0.30) for best-practice life expectancy. According to our forecasts, and in contrast to the first prediction, none of the cohorts considered are projected to attain a life expectancy of 100. That said, we make no claim that the forecasted pace of mortality decline will remain unchanged for cohorts beyond the scope of our analysis.

To quantify the deceleration in life expectancy gains among cohorts born between 1939 and 2000, we calculate the percentage reduction in the forecasted pace of mortality improvement relative to the historical trend. Our results apply exclusively to the cohorts and countries included in the analysis. In this sense, the slower pace of improvement of 0.29 y per cohort is consequential, as it would reduce the gains in cohort life expectancy by 37% (1 - 0.29/0.46) relative to the gains observed in 1900–1938. For the median, a deceleration is also evident, with a gain of 0.23 y (95% PI: 0.22, 0.23), reducing the pace of mortality improvements by 52% (1 - 0.23/0.48) with respect to the historical pace.

The results from the five additional methods are presented in Table 1. They suggest that the forecasted deceleration is not exclusive to the CPS approach; instead, all methods forecast a reduction in the pace of improvement in cohort life expectancy for both the best-practice and median cases. Furthermore, across all methods, the optimistic scenario consistently exceeds the upper PI. The forecasted pace of improvement in best-practice cohort life expectancy ranges from 0.29 y per cohort (95% PI: 0.28, 0.30), according to the CPS approach, to 0.22 y per cohort (95% PI: 0.18, 0.28) based on LLC forecasts. In turn, the widely used 2024 World Population Prospects (20) forecasts a pace of 0.23 y per cohort. The forecasted slow down is impactful; as a result, the percentage reduction of the best-practice pace of mortality improvements is 50% relative to the optimistic scenario. While the specific results vary by method, the case of the median shows similar outcomes, with gains ranging from 0.27 y per cohort (95% PI: 0.22, 0.23) to 0.20 y per cohort. Taken together, these findings suggest that all the forecasts indicate a deceleration. Forecasted cohort life expectancy improvements range from 37% to 52% of the best-practice pace, and 44% to 58% of the median.

**Table 1.** Linear trajectories over mortality forecasting methods

Method	Best-practice	Median	% Reduction (BP)	% Reduction (Med)
Extrapolation	0.46	0.48		
C-STAD	0.28 (0.22, 0.37)	0.27 (0.2, 0.33)	39%	44%
CPS	0.29 (0.28, 0.3)	0.23 (0.22, 0.23)	37%	52%
CoDa	0.24 (0.22, 0.27)	0.27 (0.24, 0.29)	48%	44%
LC	0.27 (0.25, 0.3)	0.22 (0.2, 0.25)	41%	54%
LLC	0.22 (0.18, 0.28)	0.23 (0.22, 0.24)	52%	52%
WPP2024	0.23	0.2	50%	58%

Slopes comparison across mortality forecasting methods and the percentage reduction for best-practice (BP) and median (Med) scenarios. Slopes for 95% PI in parenthesis.

### Robustness.

Does the deceleration implied by the forecasts represent a plausible trend, or could it be a result of systematic underestimation of cohort life expectancy improvements by these methods? Previous results indicate that, in some instances, some methods may have suffered from a downward bias in some similar applications (23–25). To assess this possibility, we forecast cohort life expectancy for the 20 cohorts born between 1919 and 1938, for which observed data is available, using the same methods (except for the WPP method, which is not available for the period). This period, which immediately precedes the one considered in our main analysis, represents a time during which the pace of cohort life expectancy gains in the optimistic scenario held steadily.

**Fig. 2.**
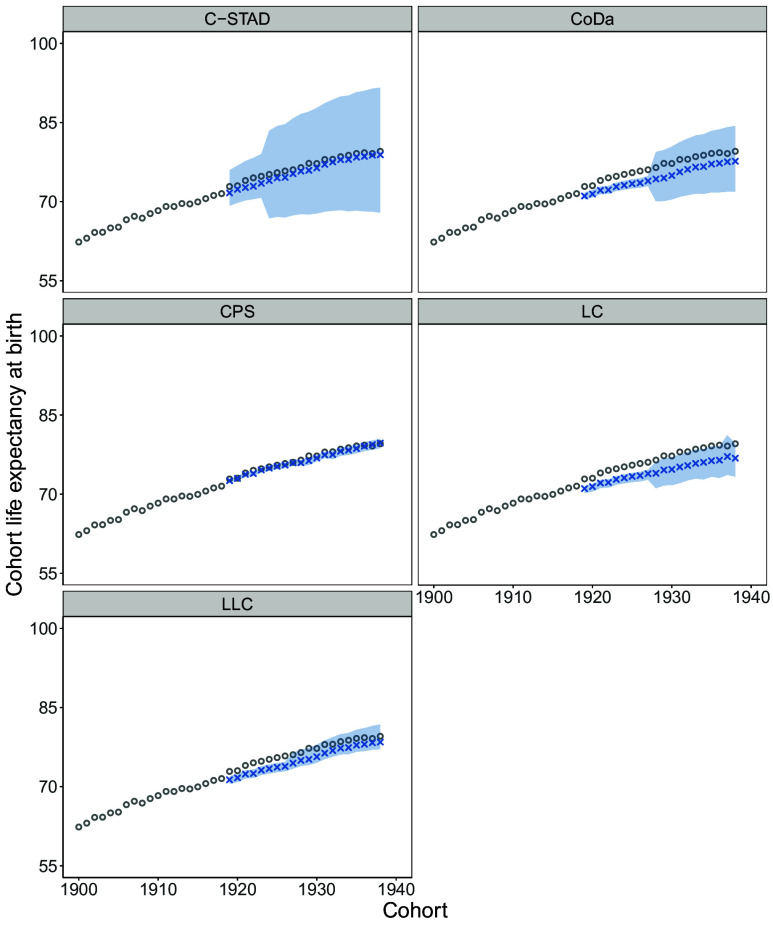
Best-practice cohort life expectancy observed (gray solid dots) in 1900–1938 compared with the best-practice cohort life expectancy forecasted (blue cross) in 1919–1938 over mortality forecasting methods.

Fig. 2 displays the forecasted (blue) and observed (gray) cohort life expectancy, along with 95% bootstrapped (PIs, shown as the shaded area), for all available methods in the best-practice case (the median is presented in *SI Appendix*, Fig. S1). A graphical examination suggests that some methods, such as LC, LLC, and CoDa, tend to underestimate cohort life expectancy gains, whereas others, such as C-STAD, and CPS, show no consistent underestimation patterns. Nonetheless, for most methods, observed cohort life expectancy falls within their respective prediction intervals for most, if not all, cohorts.

While some methods appear to underestimate cohort life expectancy for those born between 1919 and 1938, we assess whether this downward bias is large enough to explain the deceleration forecasted by these same methods for cohorts born after 1938. To do so, we first calculate the size of the gap between observed and forecasted cohort life expectancy for the 1919–1938 cohorts, measured by the absolute mean deviation. We then compare this to the gap in Fig. 1, defined as the difference between the optimistic scenario and our forecasts for the 1939–1958 cohorts. The goal is to evaluate whether the observed bias could plausibly fully account for the forecasted deceleration. If the gap in Fig. 1 exceeds that in Fig. 2, it would indicate that the bias alone cannot explain our main findings.

We adopt this approach instead of comparing the pace of cohort life expectancy improvement (slope) as in Table 1 because the shorter time horizon considered in this robustness exercise results in overly sensitive linear trends. We work with 20 cohorts for two reasons: one substantive and one technical. Substantively, we focus on cohorts close in time to our forecasts to ensure their relevance. Technically, cohort methods have strong data requirements, which limit the range of cohorts we can analyze.

The results from this exercise are summarized in Table 2. Table 2 presents the absolute mean deviation in cohort life expectancy between the observed and forecasted values shown in Fig. 2 for the best-practice case (column 3) and the median case (column 5). Additionally, it includes the same measure for the gap between the optimistic and forecasted scenarios from the main results (Fig. 1 and Table 1) for the first 20 cohorts, for comparability.

**Table 2.** Cohort life expectancy absolute mean deviation

Method	1939–1958 (BP)	1919–1938 (BP)	1939–1958 (Med)	1919–1938 (Med)
C-STAD	2.98 (2.07, 3.82)	0.92 (8.31, 8.88)	0.79 (0.51, 1.91)	0.86 (2.95, 12.6)
CPS	2.75 (2.67, 2.83)	0.36 (0.29, 0.98)	2.26 (2.19, 2.35)	1.33 (1.23, 1.92)
CoDa	2.85 (2.55, 3.15)	2.06 (2.66, 5.16)	2.04 (1.8, 2.27)	0.98 (1.58, 2.01)
LC	3.01 (2.58, 3.65)	2.37 (0.87, 4.51)	2.10 (1.64, 2.72)	1.31 (0.69, 3.17)
LLC	3.62 (3.45, 3.80)	1.53 (1.12, 2.57)	2.00 (1.67, 2.32)	0.69 (1.09, 1.45)
WPP 2024	4.31		2.53	

Absolute mean deviation of cohort life expectancy for the observed and forecasted values and the best-practice (BP) and the median (Med) scenario. Absolute mean deviation for 95% PI in parenthesis.

At one extreme, the LC method underestimates cohort life expectancy by an average of 2.37 y (95% PI: 0.87, 4.51) for the cohorts shown in Fig. 2 (Table 2, column 2). A similar comparison under the optimistic scenario yields a comparable gap of 3.01 y (95% PI: 2.58, 3.65) for the same method (Table 2, column 1). In other words, the gap for the observed cohorts is 79% (2.37/3.01) of the gap for the forecasted cohorts. By contrast, the CPS forecasts deviate by only 0.36 y (95% PI: 0.29, 0.98) from the observed average, including both over- and underestimation of actual cohort life expectancy, and show a larger gap of 2.75 y (95% PI: 2.67, 2.83) in the main results. Thus, the observed gap is just 13% (0.36/2.75) of the forecasted gap. Using the median instead of the mean yields similar results. Overall, this comparison shows that the gaps in the main results are consistently larger than those observed in Fig. 2 across all methods. The downward bias in forecasts for previously observed cohorts accounted for 13 to 79% of the divergence from optimistic scenarios under the best-practice forecasts, and 34 to 62% under the median scenario forecasts, with the exception of the more imprecisely estimated C-STAD model. Altogether, these findings indicate that the deceleration observed in our main results (Fig. 1) is unlikely to be driven solely by systematic downward bias in the forecasting methods used.

### Evaluation.

What drives the forecasted deceleration in the pace of mortality improvement? To address this question, we present two additional sets of results. First, we compare the forecasted trends in age-specific mortality to the prior trends. Next, we assess the extent to which these trends have contributed to the deceleration in cohort life expectancy for the 1939+ cohorts.

**Fig. 3.**
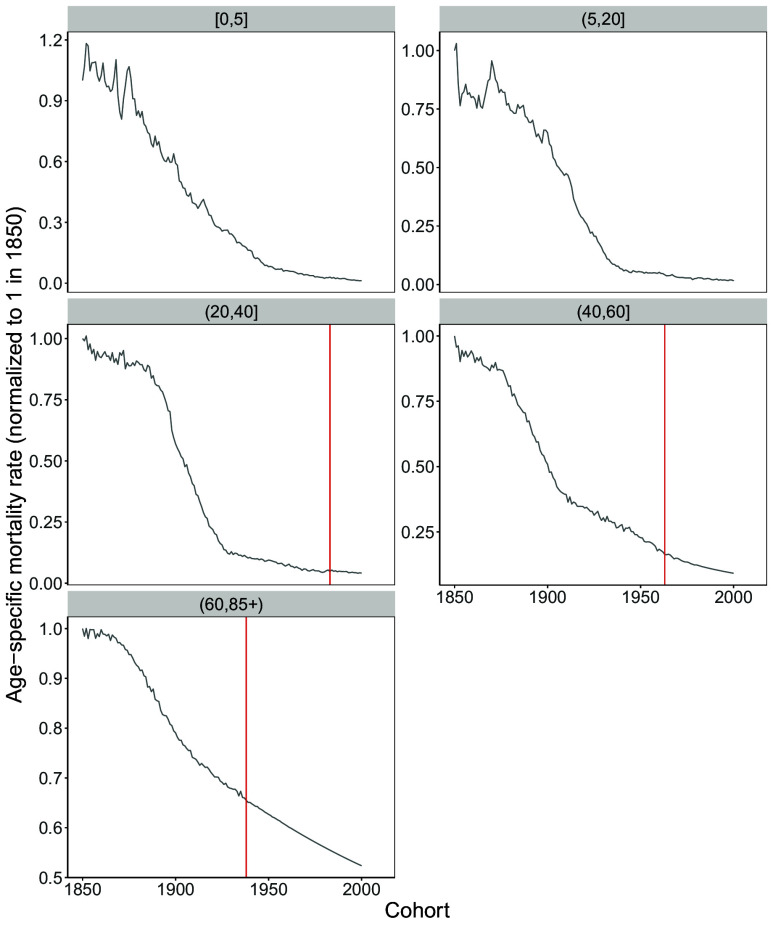
Cohort mortality rates trends in Sweden, normalized to 1 in 1850 over age groups. Before the solid line (red) all values are observed, after the line the values are forecasted (using the CPS method).

Do the forecasted mortality trends represent a break from historical trajectories? Fig. 3 illustrates the trends in mortality rates across five age groups for Sweden, with values normalized to 1 in 1850. Sweden is chosen due to its consistent data availability across the entire study period. These trends include both historical mortality rates and forecasts based on the CPS approach. The vertical line, starting in the third panel (ages 20 to 40), marks the first cohort for which mortality is forecasted in this age group. Since the youngest cohort in our analysis is age 20, age-specific mortality for the first two age groups reflects only historical data for all cohorts, whereas forecasted mortality is progressively incorporated for more recent cohorts in the last three age groups. Additionally, the forecasted trends appear to represent a relatively smooth continuation of long-standing historical trajectories across all age groups, with no evidence of structural breaks. This pattern also holds for other countries and forecasting methods; the best-practice and median cases are reported in *SI Appendix* for all methods and age groups.

What age groups drive the forecasted deceleration in cohort life expectancy for cohorts born in 1939 and later? Although all age groups exhibit a slowdown in mortality improvements, we quantify their contributions to this deceleration through an age decomposition analysis (results for single ages are available in *SI Appendix*, Table S5). Specifically, we compare age-specific gains across two sets of cohorts in two distinct periods. The first set includes cohorts born between 1900 and 1938, which provide the basis for calculating the pace of improvement in cohort life expectancy under the optimistic scenario. The second set consists of cohorts included in our forecasts, born between 1939 and 2000. For each period, we apply Arriaga’s method (26) to decompose the age-specific contributions to changes in cohort life expectancy across contiguous cohorts. For example, cohort life expectancy increased by 1.08 y between the 1901 and 1902 cohorts, with 0.40 y attributable to mortality improvements at ages 0 to 5, 0.13 y at 5 to 20, 0.21 y at 20 to 40, 0.15 y at 40 to 60, and 0.20 y at 60 to 85+. We then calculate the average contribution across all pairs of cohorts (additional details in *Materials and Methods* and *SI Appendix*).

Table 3 presents the results of this exercise for Switzerland, a country frequently identified across methods as best-practice. The findings are robust to the choice of country, as shown in the results for additional countries presented in *SI Appendix*, Table S6. Of the 0.32-y difference in the average gain between the two periods, 0.17 y, or 54% of the total, is explained by the reduction in gains for the youngest age group (ages 0 to 5). Combined with the second youngest age group (ages 5 to 20), these younger ages account for 70% of the differences in the pace of cohort life expectancy improvements across the two periods. Importantly, these are age groups for which mortality is observed, not forecasted, for all cohorts. The remaining age groups also exhibit a slowdown, though their contributions to the differences in life expectancy gains are less pronounced, particularly for the oldest age group, which accounts for only 5% of the difference. For instance, cohort life expectancy is forecasted to increase by 0.22 y between the 1964 and 1965 cohorts, with 0.02 y attributable to mortality improvements at ages 0 to 5, 0.02 y at 5 to 20, 0.001 y at 20 to 40, 0.07 y at 40 to 60, and 0.10 y at 60 to 85+. These results indicate that, generally, the deceleration in cohort mortality improvements is not a new phenomenon but rather the outcome of already observed mortality trends. Specifically, the gains at younger ages, which previously drove steady increases in cohort life expectancy, have been largely exhausted.

**Table 3.** Average contributions of changes in cohort life expectancy

Age group	1900–1938	1939–2000	Difference	Difference (%)	Difference (cum %)
Total	0.52	0.2 (0.32)	0.32 (0.19)		
[0,5]	0.24	0.07 (0.07)	0.17 (0.17)	0.54	0.54
(5,20]	0.06	0.01 (0.01)	0.05 (0.05)	0.16	0.70
(20,40]	0.07	0.01 (0.01)	0.06 (0.06)	0.18	0.88
(40,60]	0.04	0.02 (0.03)	0.02 (0.02)	0.07	0.95
(60,85+)	0.11	0.09 (0.21)	0.02 (−0.1)	0.05	1.00

Average contributions to changes in cohort life expectancy across contiguous cohorts in Switzerland, using CPS forecasts. Contributions are shown by group of cohorts and age group, along with absolute, percentage, and cumulative percentage differences. Values in parentheses indicate results under a scenario where the pace of mortality decline is doubled relative to the original forecast.

Could the forecasted deceleration be reversed if mortality improvements exceed our forecasts? To assess this, we recalculate future mortality improvements under the assumption that our forecasts underestimate mortality reductions by a factor of two across all age groups. Table 3 presents, in parentheses, the pace of improvement resulting from mortality reductions occurring at twice the rate of our CPS forecasts (additional scenarios are detailed in *SI Appendix*, Table S7).

Even under this more optimistic scenario, we continue to find substantial deceleration. Doubling future mortality gains raises the average improvement rate from 0.2 to 0.32 y per cohort for currently alive cohorts (1939–2000), still markedly below the pace observed in the preceding period (0.46 y). The oldest age group (60 to 85+) primarily drives this accelerated improvement, while younger age groups contribute only marginally. This occurs because most cohorts in our analysis have yet to reach these advanced ages, allowing future gains at older ages to exert greater influence. Under this scenario, mortality gains at older ages nearly double those observed in previous cohorts (0.21 compared to 0.11 for pre-1938 cohorts). Nevertheless, even these extreme future improvements cannot fully compensate for the exhaustion of mortality gains at younger ages, which previously drove the historical pace of improvement and whose limits are already evident in observed mortality trends.

## Discussion

A wide range of forecasting approaches suggests a significant deceleration in the pace of cohort life expectancy improvements for currently living cohorts, disrupting the long-standing trend of steady gains observed over the past 39 cohorts. Nonetheless, the magnitude of the forecasted deceleration varies, representing a reduction of 37% to 52% from the best practice pace of 0.46 y per cohort observed in the 1900–1938 cohorts, depending on the method used to complete cohort mortality. The deceleration in the median forecast ranges from 44% to 58%, further indicating that this is a widespread phenomenon, not limited to the leading countries. As a result, none of the 1939–2000 cohorts are forecasted to reach a life expectancy of 100.

Forecasting exercises are inherently uncertain, and there is no guarantee that mortality trends will remain uninterrupted. Unpredictable events—such as epidemics, social crises like U.S. deaths of despair (27), or medical breakthroughs—can cause mortality to deviate significantly from forecasted trends (28). Nevertheless, our findings suggest that the current deceleration is largely attributable to the slower pace of improvement at very young ages, a development that has already occurred for all the cohorts included in our analysis.

The primary driver of previous life expectancy gains, improvements at younger ages, appears to be approaching a lower bound in many countries within our sample. This raises a critical question: What potential exists for future positive developments to reverse this deceleration? Targeting mortality improvements at middle ages could yield substantial gains in life expectancy (29). Additionally, advancements in medical science-particularly those addressing the underlying causes of age-related mortality and improving behavioral risk factors-could significantly delay the onset of aging in humans (8, 30, 31). However, our results suggest that, at least for currently living cohorts, even substantial new mortality gains are unlikely to reverse the forecasted deceleration in life expectancy.

The findings of this study are not intended to be interpreted as evidence in favor or against a biological age limit to human life. Instead, the observed deceleration in cohort life expectancy is likely influenced by a combination of biological and social determinants, as highlighted in previous research (4, 8, 32). Existing work has identified a slowdown in period life expectancy in the United States and other developed nations (15, 16), a stagnation that reflects deeper economic and social factors that underscore the complex interplay between societal conditions and health outcomes (33, 34). Our results suggest that this slow down is not only a period phenomenon but also manifests at the cohort level, indicating a broader deceleration in the pace of gains in human longevity.

## Materials and Methods

### Data.

Age-specific mortality rates are sourced from the Human Mortality Database (22). The countries included in our sample align with those analyzed by Shkolnikov et al. (6) and Jdanov and Jasilionis (3) and are detailed in *SI Appendix*, Table S2.

### Forecasts.

Cohort mortality is forecasted up to age 100+ to enhance accuracy; however, for consistency with existing literature (3), life expectancy is calculated using a life table with an open-age interval of 85+, following standard demographic methods (35). This choice has minimal impact on life expectancy at birth. The analysis includes only cohorts aged 20 or older to ensure sufficient observed data for reliable forecasting.

Two main approaches are used to complete cohort mortality profiles: period-based forecasting-which extracts the Lexis diagonal to approximate cohort mortality-including methods such as Lee–Carter (17), CP-Splines (18), CoDa (19), and the UN WPP medium scenario (20); and cohort-specific methods, including Linear Lee–Carter and C-STAD (21). Descriptions of each method are provided in *SI Appendix*, section 1D, and the corresponding training sets are listed in *SI Appendix*, Table S3. Japan is excluded from the CoDa country sample due to implausible forecast results (*SI Appendix*, Fig. S8).

PIs at the 95% level are constructed using bootstrap methods using 100 iterations. For the best-practice and median cases shown in Figs. 1 and 2 and Tables 1 and 2, the PIs reported are specific to the country holding best-practice or median status for the respective analysis. No prediction intervals are available for the WPP estimates.

### Age decomposition.

Changes in cohort life expectancy are decomposed into age-specific contributions for contiguous cohorts within two groups: 1900–1938 and 1939–2000. The average changes and contributions are summarized in Table 3 and *SI Appendix*, Table S6, with detailed results in *SI Appendix*, Fig. S5 and Table S7. To avoid cross-country variation, Switzerland is used as a reference case. For cohorts born between 1939 and 2000, CPS-based forecasts are applied.

All code for replication can be found at: OSF repository.

## Supplementary Material

Appendix 01 (PDF)

## Data Availability

Secondary data have been deposited in Open Science Framework (OSF) (https://osf.io/d5sfu/) (36).

## References

[1] V. Canudas-Romo, Three measures of longevity: Time trends and record values. Demography **47**, 299–312 (2010). 20608098 10.1353/dem.0.0098 PMC3000019

[2] A. R. Omran, The epidemiologic transition: A theory of the epidemiology of population change. Milbank Mem. Fund Q. **49**, 509 (1971). 5155251

[3] D. Jdanov, D. Jasilionis, Optimistic versus pessimistic scenarios for future life expectancy. Nat. Aging **4**, 1524–1526 (2024). 39375566 10.1038/s43587-024-00722-z

[4] J. W. Vaupel, F. Villavicencio, M. P. Bergeron-Boucher, Demographic perspectives on the rise of longevity. Proc. Natl. Acad. Sci. U.S.A. **118**, e2019536118 (2021). 33571137 10.1073/pnas.2019536118 PMC7936303

[5] T. Torri, J. W. Vaupel, Forecasting life expectancy in an international context. Int. J. Forecast. **28**, 519–531 (2012).

[6] V. M. Shkolnikov, D. A. Jdanov, E. M. Andreev, J. W. Vaupel, Steep increase in best-practice cohort life expectancy. Popul. Dev. Rev. **37**, 419–434 (2011). 22167810 10.1111/j.1728-4457.2011.00428.x

[7] J. Oeppen, J. W. Vaupel, Broken limits to life expectancy. Science **296**, 1029–1031 (2002). 12004104 10.1126/science.1069675

[8] S. J. Olshansky, B. J. Willcox, L. Demetrius, H. Beltrán-Sánchez, Implausibility of radical life extension in humans in the twenty-first century. Nat. Aging **4**, 1635–1642 (2024). 39375565 10.1038/s43587-024-00702-3 PMC11564081

[9] X. Dong, B. Milholland, J. Vijg, Evidence for a limit to human lifespan. Nature **538**, 257–259 (2016). 27706136 10.1038/nature19793 PMC11673931

[10] S. J. Olshansky, B. A. Carnes, A. Désesquelles, Prospects for human longevity. Science **291**, 1491–1492 (2001). 11234076 10.1126/science.291.5508.1491

[11] S. J. Olshansky, B. A. Carnes, C. Cassel, In search of methuselah: Estimating the upper limits to human longevity. Science **250**, 634–640 (1990). 2237414 10.1126/science.2237414

[12] J. Vallin, F. Meslé, The segmented trend line of highest life expectancies. Popul. Dev. Rev. **35**, 159–187 (2009).

[13] K. Christensen, G. Doblhammer, R. Rau, J. W. Vaupel, Ageing populations: The challenges ahead. Lancet **374**, 1196–1208 (2009). 19801098 10.1016/S0140-6736(09)61460-4 PMC2810516

[14] S. Tuljapurkar, N. Li, C. Boe, A universal pattern of mortality decline in the G7 countries. Nature **405**, 789–792 (2000). 10866199 10.1038/35015561

[15] J. B. Dowd, A. Polizzi, A. M. Tilstra, Progress stalled? The uncertain future of mortality in high-income countries Popul. Dev. Rev. **51**, 257–293 (2024).

[16] J. Y. Ho, A. S. Hendi, Recent trends in life expectancy across high income countries: Retrospective observational study. BMJ **362**, k2562 (2018). 30111634 10.1136/bmj.k2562 PMC6092679

[17] R. D. Lee, L. R. Carter, Modeling and forecasting U. S. mortality. J. Am. Stat. Assoc. **87**, 659 (1992).

[18] C. G. Camarda, Smooth constrained mortality forecasting. Demogr. Res. **41**, 1091–1130 (2019).

[19] M. P. Bergeron-Boucher, V. Canudas-Romo, J. E. Oeppen, J. W. Vaupel, Coherent forecasts of mortality with compositional data analysis. Demogr. Res. **37**, 527–566 (2017).

[20] United Nations Development Programme, World population prospects 2024: Data sources (2024). https://population.un.org/wpp/. Accessed 18 December 2024.

[21] U. Basellini, S. Kjærgaard, C. G. Camarda, An age-at-death distribution approach to forecast cohort mortality. Insur. Math. Econ. **91**, 129–143 (2020).

[22] Human Mortality Database, Berkeley, CA (USA): University of California, Berkeley; Rostock, Germany: Max planck institute for demographic research (2022). www.mortality.org. Accessed 18 December 2024.

[23] H. L. Shang, H. Booth, R. Hyndman, Point and interval forecasts of mortality rates and life expectancy: A comparison of ten principal component methods. Demogr. Res. **25**, 173–214 (2011).

[24] H. Booth, R. J. Hyndman, L. Tickle, P. De Jong, Lee-carter mortality forecasting: A multi-country comparison of variants and extensions. Demogr. Res. **15**, 289–310 (2006).

[25] R. Lee, T. Miller, Evaluating the performance of the lee-carter method for forecasting mortality. Demography **38**, 537–549 (2001). 11723950 10.1353/dem.2001.0036

[26] E. E. Arriaga, Measuring and explaining the change in life expectancies. Demography **21**, 83–96 (1984). 6714492

[27] A. Case, A. Deaton, Deaths of Despair and the Future of Capitalism (Princeton University Press, 2020).

[28] S. Schnürch, T. Kleinow, R. Korn, A. Wagner, The impact of mortality shocks on modelling and insurance valuation as exemplified by COVID-19. Ann. Actuar. Sci. **16**, 498–526 (2022).

[29] J. Flecther, J. Shi, Trend breaks in us life expectancy over 120 years and potential sources of future gains. *Popul. Stud.*, in press. 10.1080/00324728.2025.2546993 40905144

[30] H. R. Warner, F. Sierra, The longevity dividend: Why invest in basic aging research? Can. J. Aging **28**, 391–394 (2009). 20166274 10.1017/s0714980809990286

[31] S. J. Olshansky, B. A. Carnes, Primary prevention with a capital P. Perspect. Biol. Med. **60**, 478–496 (2017). 29576558 10.1353/pbm.2017.0037

[32] R. Lindahl-Jacobsen , Rise, stagnation, and rise of Danish women’s life expectancy. Proc. Natl. Acad. Sci. U.S.A. **113**, 4015–4020 (2016). 27035998 10.1073/pnas.1602783113 PMC4839462

[33] A. S. Hendi, Trends in U.S. life expectancy gradients: The role of changing educational composition. Int. J. Epidemiol. **44**, 946–955 (2015). 25939662 10.1093/ije/dyv062 PMC4607744

[34] A. Case, A. Deaton, Life expectancy in adulthood is falling for those without a BA degree, but as educational gaps have widened, racial gaps have narrowed. Proc. Natl. Acad. Sci. U.S.A. **118**, e2024777118 (2021). 33836611 10.1073/pnas.2024777118 PMC7980407

[35] S. Preston, P. Heuveline, M. Guillot, Demography: Measuring and Modeling Population Processes (Malden, MA: Blackwell Publishers, 2001).

[36] J. Andrade, C. G. Camarda, H. Pifarré i Arolas, Replication materials for: Cohort mortality forecasts indicate signs of deceleration in life expectancy gains. Open Science Framework (OSF). https://osf.io/d5sfu/. Deposited 12 July 2025. 10.1073/pnas.2519179122 PMC12415247 40854133

