# Supplementary material for: Cohort mortality forecasts indicate signs of deceleration in life expectancy gains
Source: Proc Natl Acad Sci U S A. 2025 Aug 25;122(35):e2519179122. doi: 10.1073/pnas.2519179122 (PMC12415247; doi:10.1073/pnas.2519179122)
Supplement: Supplementary file 1 — Appendix 01 (PDF) [file pnas.2519179122.sapp.pdf]

Supporting Information for

Cohort mortality forecasts indicate signs of deceleration in life expectancy gains

José Andrade, Carlo Giovanni Camarda, and Héctor Pifarré i Arolas

José Andrade.
E-mail: andrade@demogr.mpg.de

This PDF file includes:

Figs. S1 to S17
Tables S1 to S7
SI References

Contents

1 Forecasting **3**

A Forecasting results 3

B Mortality forecasting implausibility 11

C Country sample, best-practice, and median cases 12

D Forecasting methods description 15

2 Robustness **16**

A Robustness for median case 16

3 Evaluation **17**

A Contributors to cohort life expectancy deceleration and future mortality improvement scenarios 17

B Age-specific mortality rates trends 27

1. Forecasting

A. Forecasting results. This section describes the forecasted best-practice cohort mortality rates, expressed in terms of cohort mortality improvement rates by age group, as shown in Table S1. We compute these rates for two cohort groups: (i) fully observed cohorts born between 1900 and 1938 (column 2 in Table S1) and (ii) cohorts currently alive projected using different forecasting methods, born between 1939 and 2000 (columns 3–8 in Table S1). The cohort mortality improvement rate for each age group is estimated as the slope of the linear mortality trend.

$$roi_x = \frac{(m_{x2} - m_{x1})}{(cohort_2 - cohort_1)}. \quad [1]$$

Here, roi_x represents the cohort rate of improvement for age group x. The terms m_{x1} and m_{x2} denote the initial and final cohort mortality rates during the analyzed period, respectively, while $cohort_1$ and $cohort_2$ correspond to the initial and final cohorts within the same period. To enhance readability, the roi_x values have been rescaled to reflect rates per 100,000.

The cohort mortality rates of improvement are illustrated in Fig. S1 for Sweden. The solid black trend represents the observed cohort age-group mortality rates, while the colored trends correspond to the forecasted cohort age-group mortality rates. The red dashed line indicates the transition between observed (left) and forecasted (right) age-group mortality rates, as determined by the forecasting method.

Fig. S2 to Fig. S7 display the observed and forecasted trends of best-practice logged age-specific cohort mortality rates for each forecasting method. The black dashed line separates the observed logged age-specific mortality rates (left) from the forecasted ones (right). Notably, spikes in cohort age-specific mortality rates arise due to shifts in the best-practice country from one year to another. The discontinuity in the mortality trend under the UN WPP 2024 method (Fig. S7) results from data availability beginning in 1950.

Table S1. Best-practice observed and forecasted cohort mortality rates of improvement (in 100,000)

Age group	Fully obs.	C-STAD	CPS	CoDa	LC	LLC	WPP2024
[0,5]	-36.543	-14.853	-11.679	-11.496	-11.679	-13.266	-8.245
(5,20]	-10.064	-0.766	-0.716	-0.711	-0.716	-0.719	-0.641
(20,40]	-9.525	-0.762	-0.939	-0.928	-0.939	-0.772	-0.714
(40,60]	-5.466	-3.719	-3.812	-3.674	-3.733	-2.903	-2.843
(60,85+)	-26.179	-18.358	-19.111	-16.369	-17.695	-13.461	-17.227

Best-practice fully observed cohort mortality rates of improvement from 1900–1938 by age group (column 2) and best-practice forecasted cohort mortality rates of improvement from 1939–2000 by age group for each forecasting method (columns 3–8).

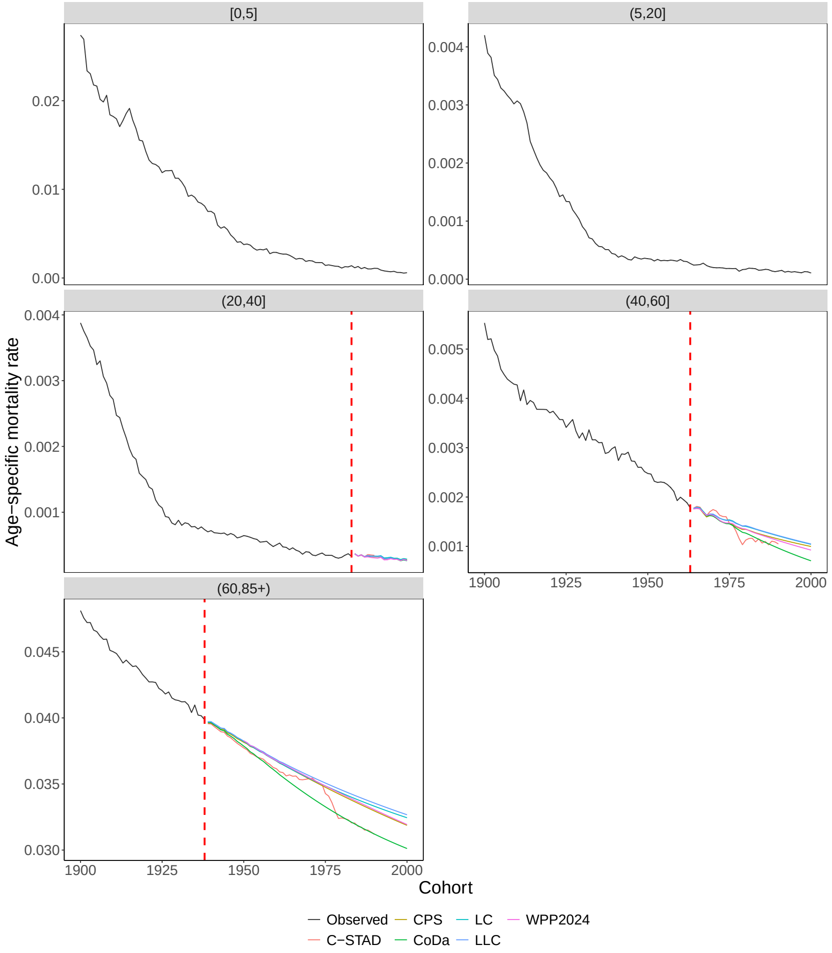

Fig. S1. Observed and forecasted age-group mortality rate trends in Sweden. The red dashed line divides each panel, distinguishing observed age-group mortality rates (left) from forecasted age-group mortality rates (right).

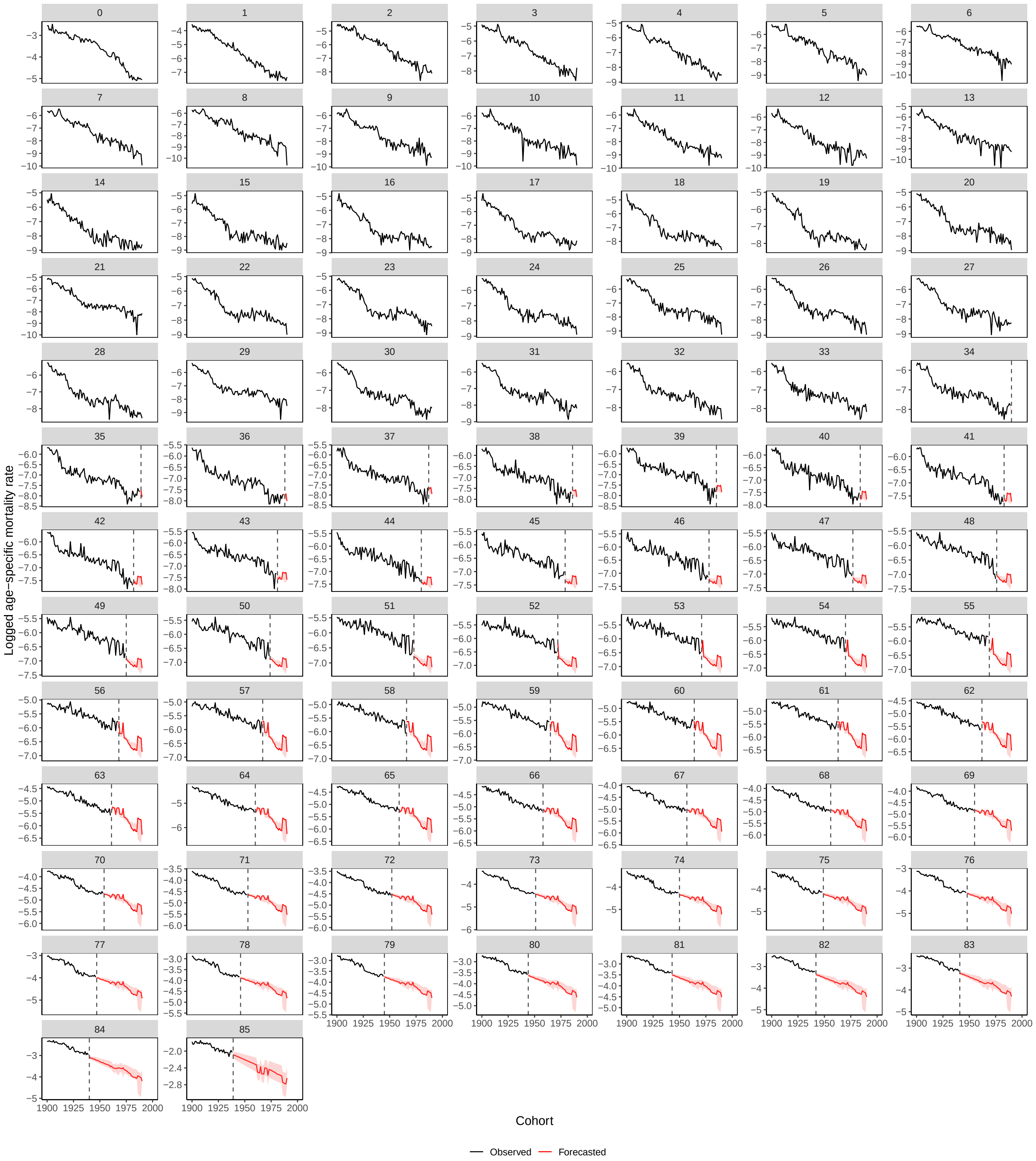

Fig. S2. Best-practice logged age-specific mortality rates trends using C-STAD method

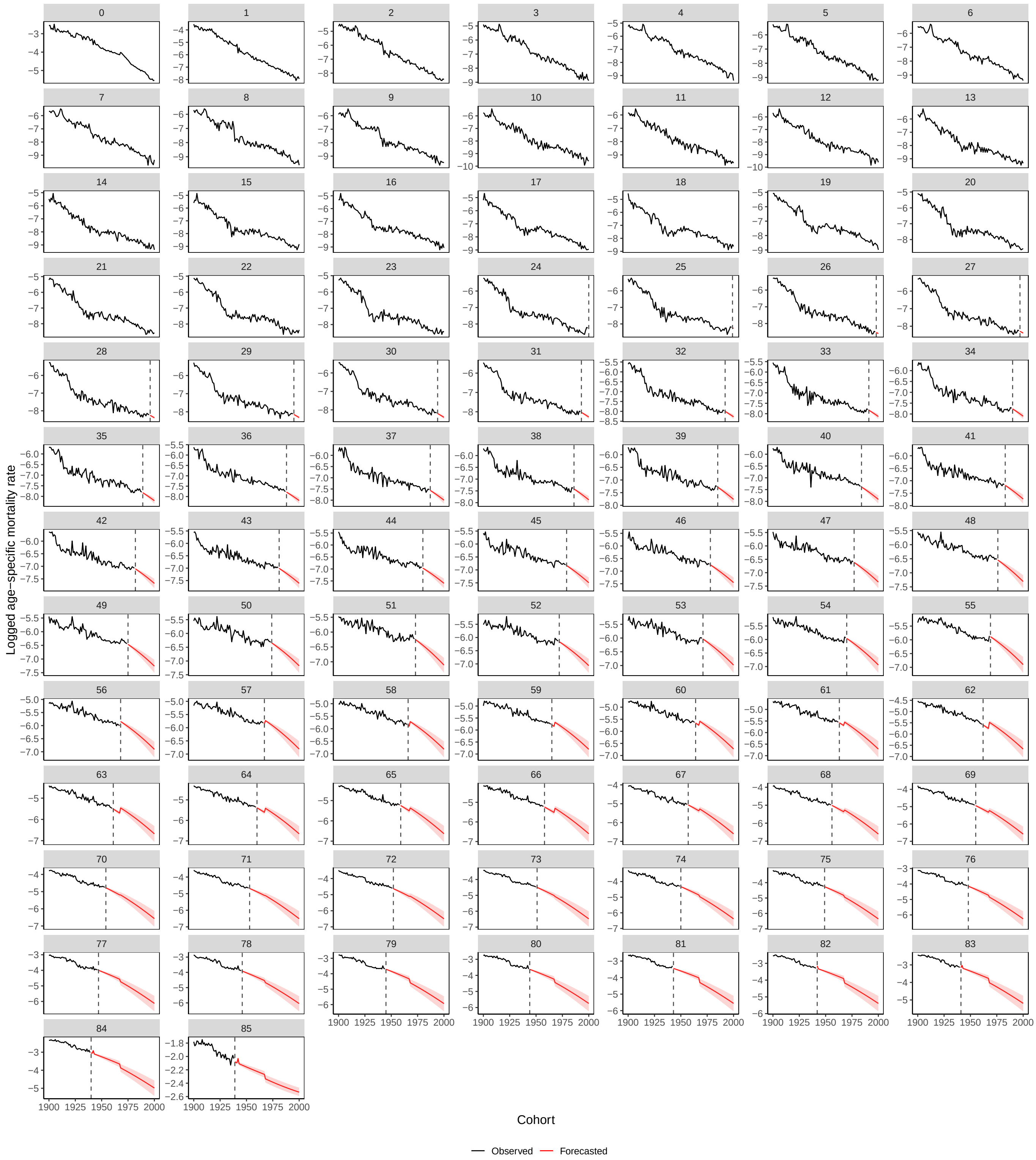

Fig. S3. Best-practice logged age-specific mortality rates trends using CoDa method

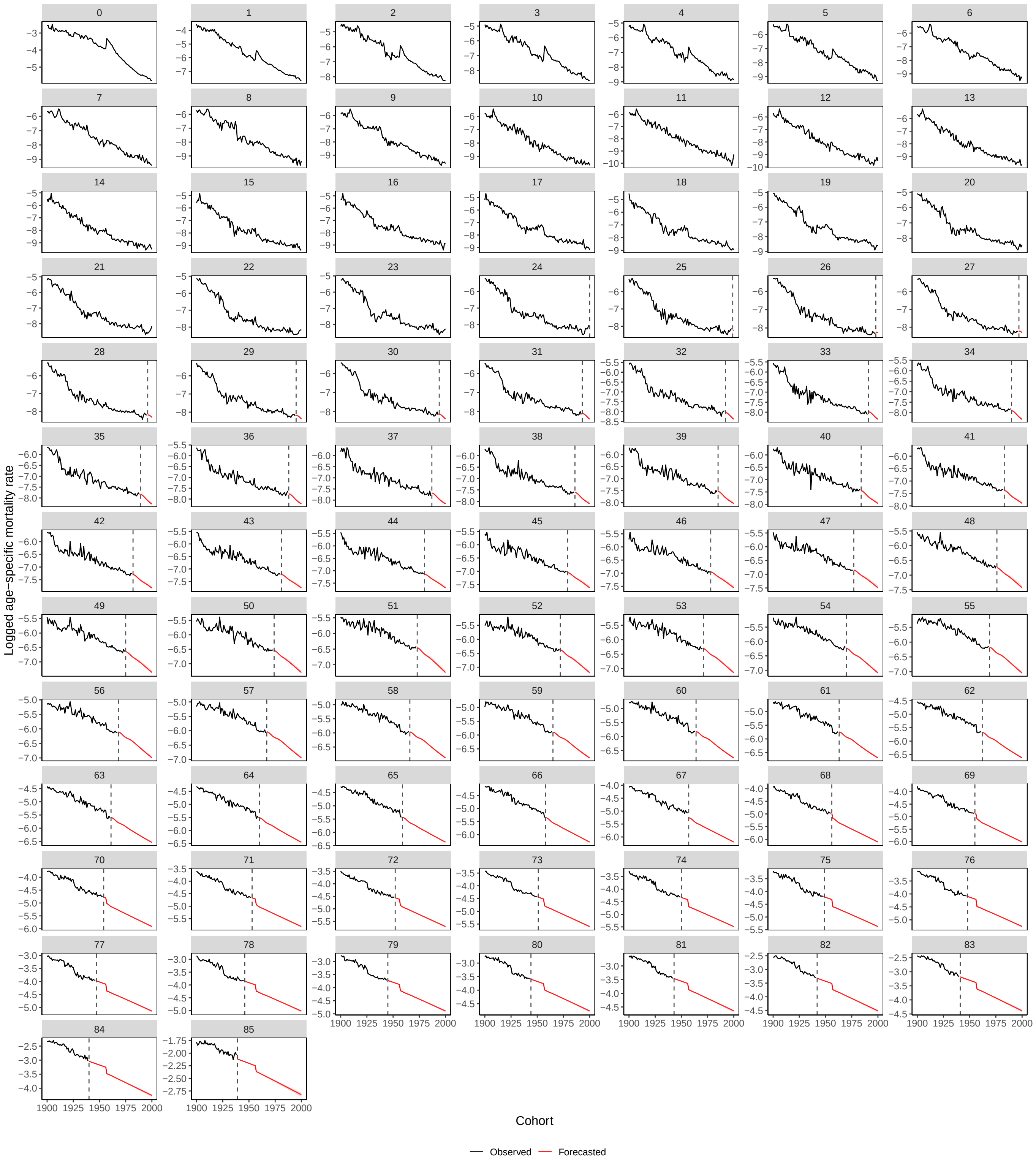

Fig. S4. Best-practice logged age-specific mortality rates trends using CPS method

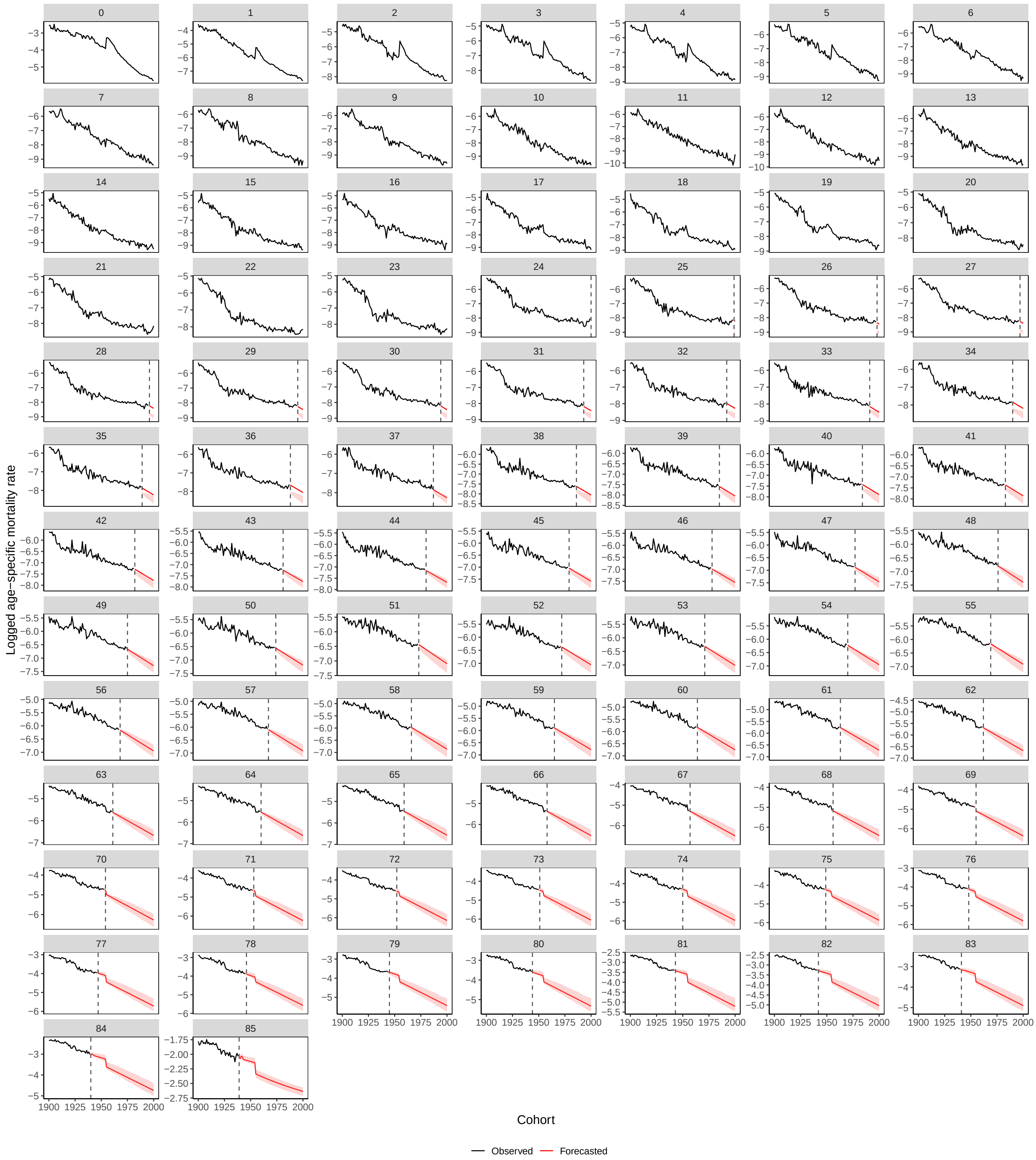

Fig. S5. Best-practice logged age-specific mortality rates trends using LC method

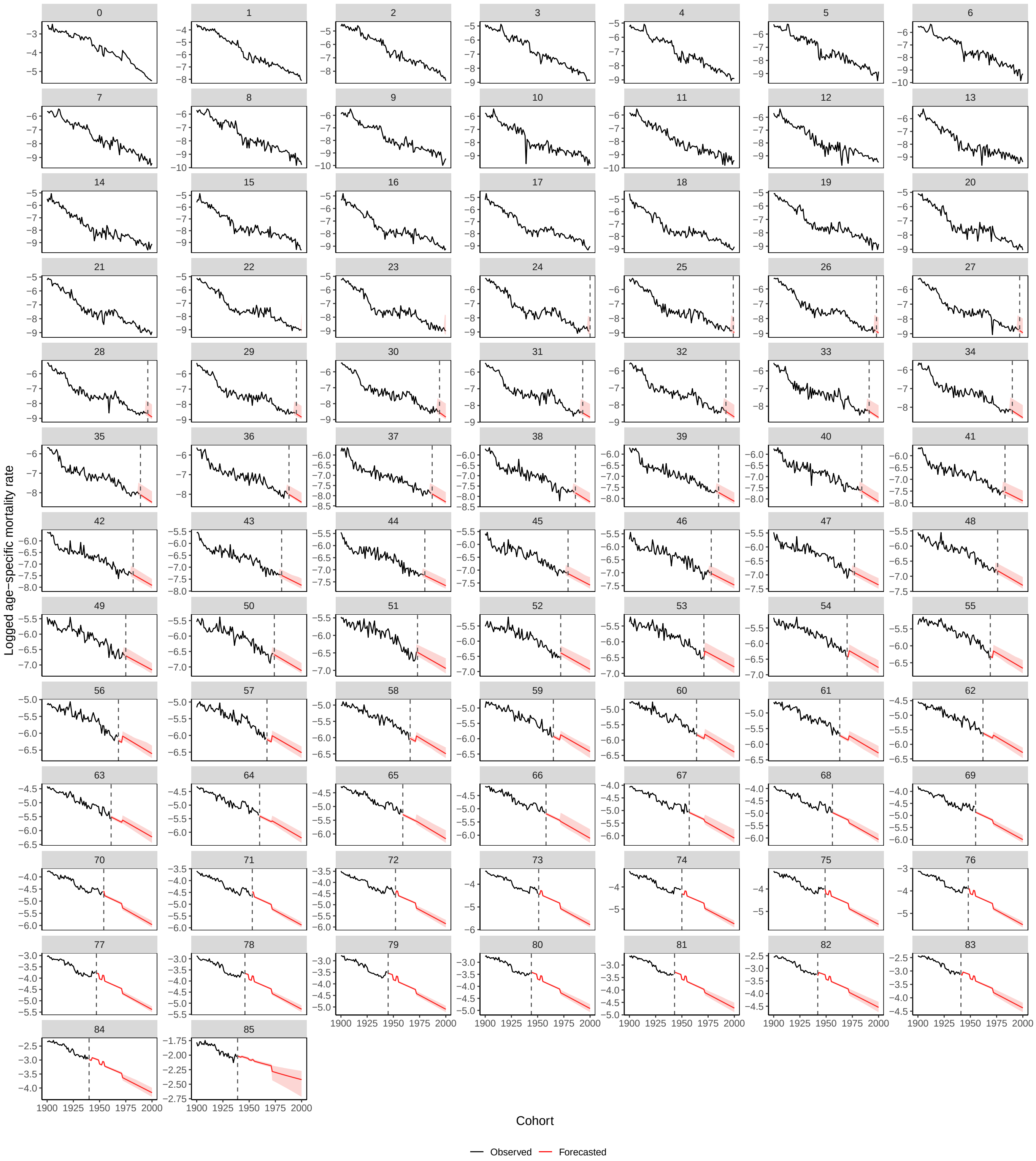

Fig. S6. Best-practice logged age-specific mortality rates trends using LLC method

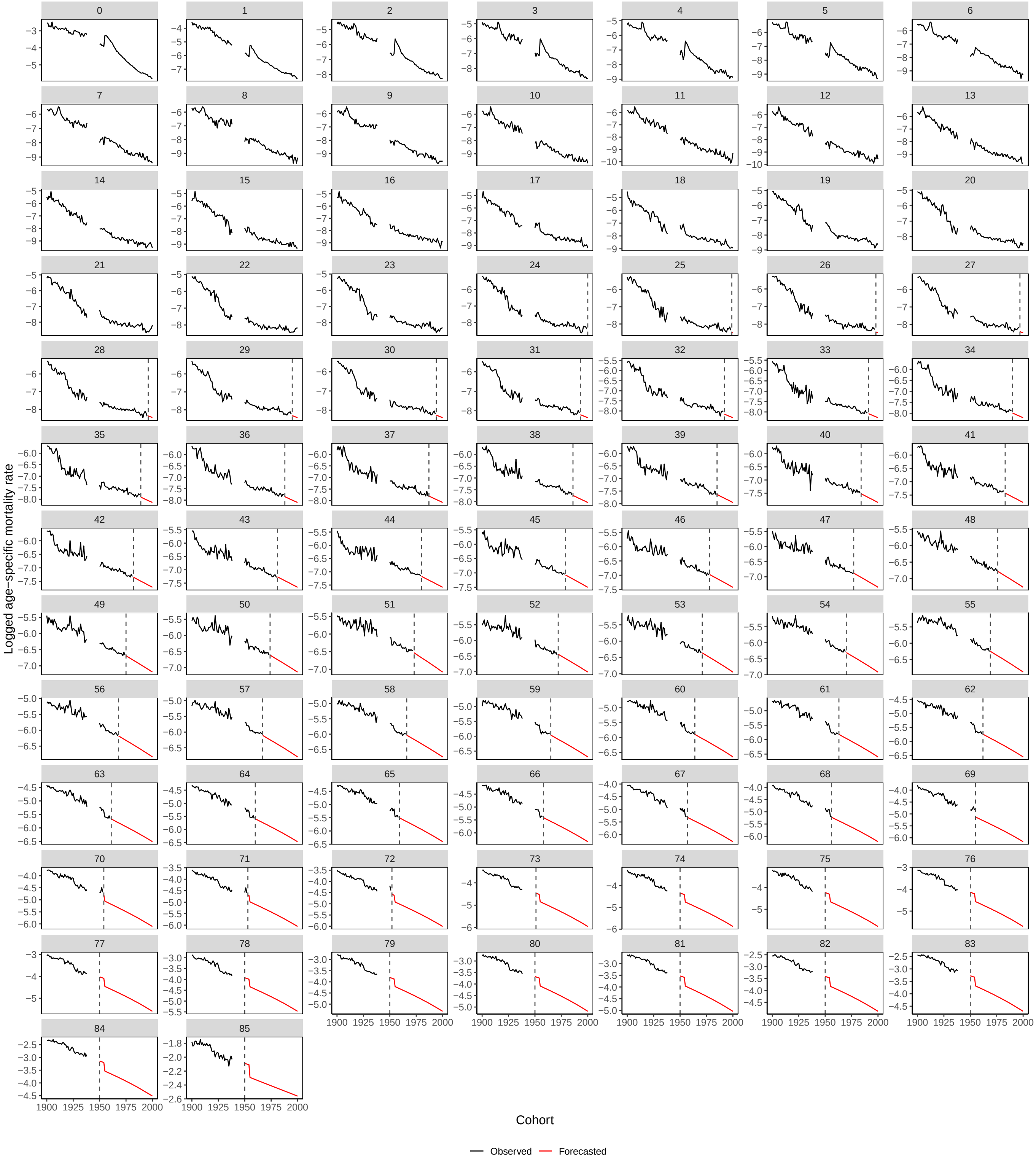

Fig. S7. Best-practice logged age-specific mortality rates trends using UN WPP 2024 method

B. Mortality forecasting implausibility. This section examines the case of mortality forecasting for Japan using the CoDa method and highlights its implausible results. Specifically, Fig. S8 displays the observed and forecasted female logged age-specific mortality rates for Japan. Given the lack of plausibility in these cohort mortality projections, this case is excluded from our analysis. Although Japan is excluded from the CoDa forecasting method due to implausible results, it remains included in the other age-period forecasting approaches, as shown in Table S3.

The CoDa approach, which forecasts mortality using compositional data based on the life table death distribution (d_x), tends to produce more optimistic projections than standard methods such as Lee–Carter. This divergence stems from CoDa's flexible modeling of mortality improvement rates (RMIs), which are not constrained to remain constant over time, in contrast to the assumptions underlying the Lee–Carter model (1). For Japan, the estimated time index in the CoDa model displays a pronounced non-linear trend. When projected forward using the standard random walk with drift approach, this trend yields implausible mortality forecasts. In particular, the extrapolated rates of mortality improvement (RMIs) at young and middle ages become exceptionally steep, producing unrealistic future age patterns of mortality. This distortion arises despite other age-specific CoDa parameters exhibiting relatively stable behavior. Together, these anomalies result in forecasts that lack plausibility, motivating the exclusion of Japan from the CoDa-based projections.

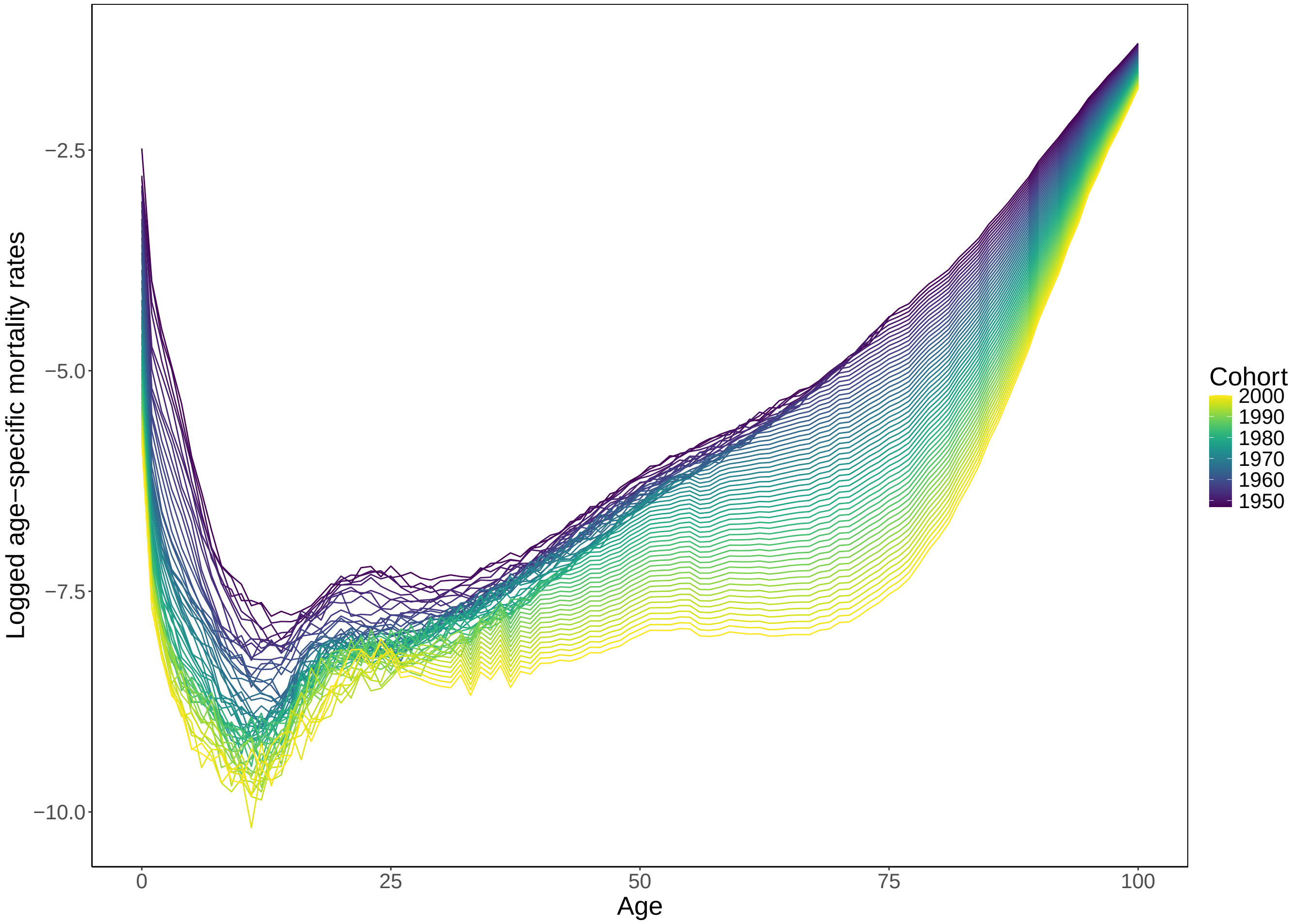

Fig. S8. Female logged age-specific mortality rates, observed and forecasted using the CoDa method in Japan

C. Country sample, best-practice, and median cases. This section provides a list of the sample of countries included in our analysis and the corresponding initial cohort identified for each country (Table S2). Additionally, Table S3 presents the training set employed to forecast cohort age-specific mortality rates across forecasting methods. Finally, Table S4 displays the best-practice and median country cases for cohorts born between 1939 and 2000.

Table S2. Countries and cohorts used in the analysis

Country code	Description	Since
AUS	Australia	1921
AUT	Austria	1947
BEL	Belgium	1919
CAN	Canada	1921
DNK	Denmark	1850
DEUTE	Germany	1956
GBRTENW	England and Wales	1850
FIN	Finland	1878
FRATNP	France	1850
ISL	Iceland	1850
IRL	Ireland	1950
ITA	Italy	1872
JPN	Japan	1947
NLD	Netherlands	1850
GBR_NIR	Northern Ireland	1922
NOR	Norway	1850
PRT	Portugal	1940
GBR_SCO	Scotland	1850
ESP	Spain	1908
SWE	Sweden	1850
CHE	Switzerland	1876
USA	United States of America	1933
DEUTW	West Germany	1956

List of countries (column 2) used in the analysis and their corresponding starting year (column 3) when data analysis begins. Column 1 shows the country code in the HMD.

Table S3. Training periods across country and mortality forecasting methods

Country	LC	CPS	CoDa	LLC	C-STAD
AUS	1950 - 2021	1950 - 2021	1950 - 2021		
AUT	1950 - 2023	1950 - 2023	1950 - 2023		
BEL	1950 - 2023	1950 - 2023		1900 - 2000	
CAN	1950 - 2022	1950 - 2022	1950 - 2022		
CHE	1950 - 2023	1950 - 2023		1900 - 2000	1900 - 2023
DEUTE	1956 - 2020	1956 - 2020	1956 - 2020		
DEUTW	1956 - 2020	1956 - 2020	1956 - 2020		
DNK	1950 - 2023	1950 - 2023	1950 - 2023	1900 - 2000	1900 - 2023
ESP	1950 - 2021	1950 - 2021	1950 - 2021	1908 - 2000	1908 - 2021
FIN	1950 - 2023	1950 - 2023		1900 - 2000	1900 - 2023
FRATNP	1950 - 2022	1950 - 2022	1950 - 2022	1900 - 2000	1900 - 2022
GBRTENW	1950 - 2022	1950 - 2022	1950 - 2022	1900 - 2000	1900 - 2022
GBR_NIR	1950 - 2022	1950 - 2022	1950 - 2022		
GBR_SCO	1950 - 2022	1950 - 2022	1950 - 2022	1900 - 2000	1900 - 2022
IRL	1950 - 2022	1950 - 2022	1950 - 2022		
ISL	1950 - 2022				
ITA	1950 - 2021	1950 - 2021	1950 - 2021	1900 - 2000	1900 - 2021
JPN	1950 - 2023	1950 - 2023			
NLD	1950 - 2022	1950 - 2022	1950 - 2022	1900 - 2000	1900 - 2022
NOR	1950 - 2023	1950 - 2023	1950 - 2023	1900 - 2000	1900 - 2023
PRT	1950 - 2023	1950 - 2023	1950 - 2023		
SWE	1950 - 2023	1950 - 2023	1950 - 2023	1900 - 2000	1900 - 2023
USA	1950 - 2022	1950 - 2022	1950 - 2022		

List of countries with the training periods used by each forecasting method to forecast cohorts that are currently alive.

Table S4. Best-practice and median countries by cohort born in 1939–2000, across forecasting methods.

Cohort	Best-practice						Median					
	C-STAD	CoDa	LC	LLC	CPS	WPP2024	C-STAD	CoDa	LC	LLC	CPS	WPP2024
1939	CHE	AUS	AUS	CHE	AUS		GBRTENW	USA	USA	DNK	USA	
1940	CHE	AUS	AUS	CHE	AUS		DNK	USA	USA	FRATNP	FRATNP	
1941	CHE	AUS	CHE	CHE	AUS		FRATNP	DNK	DNK	DNK	USA	
1942	CHE	SWE	CHE	CHE	AUS		NLD	DNK	FIN	DNK	FIN	
1943	CHE	AUS	AUS	SWE	AUS		FRATNP	FRATNP	USA	FRATNP	USA	
1944	CHE	AUS	AUS	SWE	AUS		NLD	USA	USA	FRATNP	FRATNP	
1945	CHE	AUS	AUS	SWE	AUS		FIN	GBR_NIR	FIN	NLD	FIN	
1946	CHE	AUS	AUS	SWE	AUS		FIN	DNK	FRATNP	FRATNP	FRATNP	
1947	CHE	AUS	AUS	SWE	AUS		NLD	DNK	FIN	DNK	FIN	
1948	CHE	AUS	AUS	SWE	AUS		NLD	DNK	FIN	DNK	GBR_NIR	
1949	CHE	AUS	AUS	SWE	AUS		FIN	DNK	FIN	FIN	FIN	
1950	CHE	AUS	AUS	CHE	AUS	AUS	FIN	IRL	IRL	FIN	FIN	FIN
1951	CHE	AUS	AUS	CHE	AUS	AUS	FIN	IRL	IRL	NLD	NLD	GBRTENW
1952	CHE	AUS	AUS	CHE	AUS	AUS	FIN	IRL	IRL	NLD	NLD	GBRTENW
1953	CHE	AUS	AUS	SWE	AUS	AUS	FIN	DNK	NLD	NLD	NLD	GBRTENW
1954	CHE	AUS	AUS	SWE	AUS	AUS	FIN	NLD	ESP	ESP	ESP	IRL
1955	CHE	AUS	JPN	CHE	AUS	JPN	FIN	ITA	NLD	ESP	ESP	GBRTENW
1956	CHE	AUS	JPN	CHE	AUS	JPN	FIN	ITA	CAN	ESP	NLD	IRL
1957	CHE	AUS	JPN	CHE	JPN	JPN	FIN	DNK	ESP	ESP	NLD	IRL
1958	CHE	AUS	JPN	CHE	JPN	JPN	NOR	DNK	GBRTENW	GBRTENW	DNK	NLD
1959	CHE	AUS	JPN	CHE	JPN	JPN	GBRTENW	DNK	ISL	GBRTENW	BEL	NLD
1960	CHE	AUS	JPN	CHE	JPN	JPN	NOR	NLD	BEL	GBRTENW	ITA	IRL
1961	CHE	AUS	JPN	CHE	JPN	JPN	NOR	AUT	ISL	BEL	BEL	ESP
1962	FRATNP	AUS	JPN	CHE	JPN	JPN	NOR	CAN	CAN	BEL	ITA	NLD
1963	FRATNP	AUS	JPN	CHE	JPN	JPN	NOR	GBRTENW	ISL	BEL	ITA	NLD
1964	FRATNP	AUS	JPN	CHE	JPN	JPN	GBRTENW	IRL	ITA	BEL	DEUTW	ISL
1965	CHE	AUS	JPN	CHE	JPN	JPN	GBRTENW	AUT	ITA	BEL	DEUTW	ISL
1966	FRATNP	AUS	JPN	CHE	JPN	JPN	NOR	AUT	CAN	BEL	DEUTW	ITA
1967	FRATNP	AUS	JPN	CHE	JPN	JPN	GBRTENW	IRL	AUT	BEL	FIN	ISL
1968	FRATNP	FRATNP	JPN	CHE	JPN	JPN	GBRTENW	AUT	CAN	BEL	DEUTW	ISL
1969	CHE	FRATNP	JPN	CHE	JPN	JPN	GBRTENW	IRL	CAN	BEL	DEUTW	ISL
1970	CHE	FRATNP	JPN	CHE	JPN	JPN	GBRTENW	IRL	CAN	ITA	DEUTW	ISL
1971	CHE	FRATNP	JPN	CHE	JPN	JPN	GBRTENW	AUT	CAN	ITA	DEUTW	IRL
1972	FRATNP	FRATNP	JPN	ESP	JPN	JPN	GBRTENW	AUT	CAN	ITA	DEUTW	IRL
1973	CHE	FRATNP	JPN	ESP	JPN	JPN	ESP	IRL	CAN	ITA	DNK	IRL
1974	CHE	FRATNP	JPN	ESP	JPN	JPN	SWE	AUT	CAN	ITA	DEUTW	ISL
1975	CHE	FRATNP	JPN	ESP	JPN	JPN	NOR	IRL	AUT	ITA	DEUTW	ISL
1976	CHE	FRATNP	JPN	ESP	JPN	JPN	SWE	IRL	AUT	ITA	DEUTW	IRL
1977	CHE	FRATNP	JPN	ESP	JPN	JPN	NOR	IRL	AUT	ITA	DEUTW	BEL
1978	CHE	FRATNP	JPN	ESP	JPN	JPN	NOR	IRL	AUT	NOR	FIN	AUT
1979	CHE	FRATNP	JPN	ESP	JPN	JPN	NOR	NOR	AUT	NOR	FIN	IRL
1980	CHE	FRATNP	JPN	ESP	JPN	JPN	NOR	NOR	AUT	NOR	PRT	ISL
1981	CHE	FRATNP	JPN	ESP	JPN	JPN	NOR	NOR	NOR	NOR	PRT	AUT
1982	CHE	FRATNP	JPN	ESP	JPN	JPN	NOR	PRT	NOR	NOR	NOR	IRL
1983	CHE	FRATNP	JPN	ESP	JPN	JPN	NOR	PRT	PRT	NOR	NOR	IRL
1984	CHE	FRATNP	JPN	ESP	JPN	JPN	NOR	PRT	AUT	BEL	BEL	PRT
1985	CHE	FRATNP	JPN	ESP	JPN	JPN	SWE	PRT	PRT	NOR	NOR	IRL
1986	FRATNP	FRATNP	JPN	ESP	JPN	JPN	SWE	PRT	AUT	BEL	BEL	ISL
1987	FRATNP	FRATNP	JPN	ESP	JPN	JPN	ITA	PRT	AUT	BEL	BEL	PRT
1988	FRATNP	FRATNP	JPN	ESP	JPN	JPN	SWE	PRT	AUT	BEL	BEL	NOR
1989	FRATNP	FRATNP	JPN	ESP	JPN	JPN	SWE	PRT	AUT	BEL	CAN	NOR
1990	CHE	FRATNP	JPN	ESP	JPN	JPN	NOR	PRT	AUT	SWE	CAN	NOR
1991		FRATNP	JPN	ESP	JPN	JPN		IRL	AUT	SWE	BEL	NOR
1992		FRATNP	JPN	ESP	JPN	JPN		PRT	AUT	BEL	BEL	NOR
1993		FRATNP	JPN	ESP	JPN	JPN		PRT	AUT	BEL	CAN	CAN
1994		FRATNP	JPN	ESP	JPN	JPN		PRT	AUT	BEL	CAN	CAN
1995		FRATNP	JPN	ESP	JPN	JPN		IRL	AUT	NOR	CAN	CAN
1996		FRATNP	JPN	ESP	JPN	JPN		IRL	AUT	SWE	CAN	CAN
1997		FRATNP	JPN	ESP	JPN	JPN		IRL	AUT	SWE	CAN	CAN
1998		FRATNP	JPN	ESP	JPN	JPN		IRL	AUT	SWE	CAN	CAN
1999		FRATNP	JPN	ESP	JPN	JPN		IRL	AUT	SWE	CAN	CAN
2000		FRATNP	JPN	ESP	JPN	JPN		IRL	AUT	SWE	CAN	CAN

List of countries representing the best-practice case by cohort in columns 2–7. List of countries representing the median case by cohort in columns 8–13 across forecasting methods.

D. Forecasting methods description. We employ five mortality forecasting methods: three based on an age-period perspective and two on an age-cohort perspective. In addition, we also incorporate the published UN World Population Prospects (WPP) forecasts.

Within the age-period perspective, we utilize the **Lee-Carter (LC)** method (2), which forecasts age-specific mortality trends using a functional form and projects the key parameter via standard time-series models. A similar methodology is applied in the **Compositional Data Analysis (CoDa)** method (1), which structures mortality data as age-at-death distributions, and forecasts its primary component using time-series techniques. Additionally, we implement the **Smooth Constrained Mortality Forecasting CP-Splines (CPS)** method (3), which smooths and forecasts age-specific mortality rates using a two-dimensional P-splines approach incorporating demographic constraints.

For the age-cohort perspective, we adopt the **Linear Lee-Carter (LLC)** method (4), which fits cohort-specific mortality rates based on the Lee-Carter (2) framework, and forecasts the key parameter linearly. We also apply the **Cohort Segmented Transformation Age-at-Death Distributions (CSTAD)** method (5), a relational approach that links a time-invariant standard distribution to observed and partially observed distributions.

Uncertainty: Prediction intervals for all forecasting methods are generated using bootstrapping techniques. In the case of the LC model, future sample trajectories are simulated based on the previously estimated model, and prediction intervals are derived from these simulated mortality rates.

For CPS and LLC, prediction intervals are constructed using a residual bootstrap approach following Koissi et al. (6). Specifically, bootstrapped death counts are generated by resampling deviance residuals with replacement while keeping the original exposures fixed. This enables model estimation and the derivation of bootstrapped parameters.

For the CoDa and C-STAD approaches, bootstrapped death counts based on deviance residuals serve as the primary source of uncertainty. Additionally, a second layer of uncertainty is incorporated by simulating future values of the time-series model associated with each forecasting approach.

2. Robustness

A. Robustness for median case. This section presents the median forecasted cohort life expectancy for the 20 cohorts born between 1919 and 1938, for which fully observed data is available, using all applicable methods (excluding the WPP method, which is unavailable for this period). Fig. S9 illustrates the forecasted (blue crosses) and observed (gray circles) cohort life expectancy, along with 95% bootstrapped prediction intervals (PIs) represented by the shaded area, for all available methods in the median case.

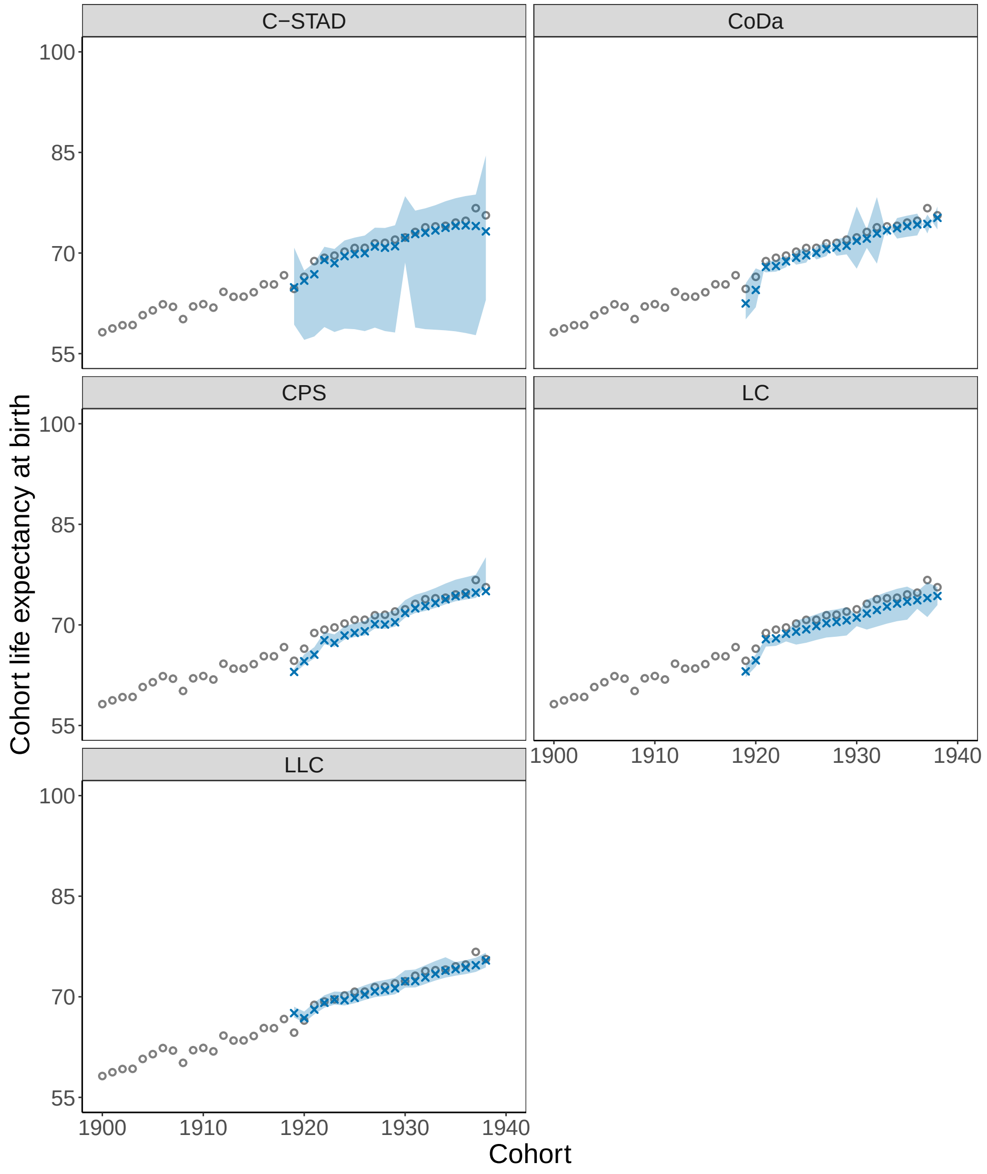

Fig. S9. Median cohort life expectancy at birth observed (gray circles) from 1900 to 1938 compared with the median cohort life expectancy at birth forecasted (blue crosses) from 1919 to 1938 across mortality forecasting methods.

3. Evaluation

A. Contributors to cohort life expectancy deceleration and future mortality improvement scenarios. This section examines the robustness of our findings under alternative mortality scenarios that deviate from our baseline forecasts. To do so, we first compute the cohort rate of improvement (ROI) using Equation 1, derived from the trend of the forecasted logged age-specific mortality rates (black dashed line in Fig. S10). The objective is to assess how modifications to the rate of improvement affect cohort life expectancy. Specifically, we systematically adjust the original forecasted ROI in increments of 0.1, ranging from half to double its initial value at ages 24–85+ (see Fig. S10). Based on these adjusted ROIs, we generate multiple scenarios for age-specific mortality rates. For each scenario, we compute the resulting changes in cohort life expectancy and decompose the contributions by age group for the 1939–2000 birth cohorts, as presented in Table 3.

Fig. S10 illustrates the trends in observed (solid black line) and forecasted (dashed black line) cohort logged age-specific mortality rates for Switzerland using the CPS forecasting method. Additionally, alternative age-specific mortality rates are generated by systematically adjusting the original forecasted ROI in increments of 0.1, ranging from half to double its initial value for ages 24–85+, represented by the gradient from yellow to red. Vertical dashed line divide the observed mortality trends (left) and the forecasted trends (right).

Fig. S11 presents the changes in cohort life expectancy from 1939 to 2000, along with the corresponding contributions by age group in Switzerland using the CPS forecasting method. This decomposition is based on scenarios in which the ROI of the original forecast is systematically adjusted from half to double its initial value for age groups 24–85+. Additionally, the black horizontal dashed line represents the average contributions to changes in cohort life expectancy for cohorts born between 1939 and 2000 under the original forecasted age-specific mortality rates (0.20, see Table 3). In contrast, the red horizontal dashed line denotes the observed average contributions for cohorts born between 1900 and 1938 (0.52, see Table 3).

Fig. S12 to Fig. S14 illustrate changes in cohort life expectancy in Switzerland when modifying age-specific mortality rates within specific age groups (i.e., 24–40, 41–60, and 61–85+, respectively). In these scenarios, only the mortality rates for a given age group are adjusted according to the simulated ROIs, while the remaining age groups retain their original forecasted mortality rates. For instance, Fig. S12 presents the changes in cohort life expectancy from 1939 to 2000, along with its age group decomposition, when adjusting mortality rates for ages 24–40 based on previously described scenarios. Similarly, Fig. S13 and Fig. S14 show the changes in cohort life expectancy when modifying mortality rates exclusively for ages 41–60 and 61–85+, respectively.

Fig. S15 displays the contiguous cohort decomposition by age in Switzerland from 1900 to 2000. Before the vertical dashed line (1938), all cohorts are fully observed; after the line, cohorts are completed using the CPS forecasting method. Average of these contributions by group of cohorts (1900–1938 and 1939–2000) are depicted in Table 3 and Table S6.

Table S6 presents the average contribution of changes in cohort life expectancy across contiguous cohorts, stratified by cohort groups and age groups (single ages decomposition are presented in Table S5). The table reports differences in average contributions, percentage differences, and cumulative percentage differences. Cohort forecasts are generated using the CPS method. Values in parentheses indicate changes and contributions to cohort life expectancy (column 3) and differences across periods (column 4) under two alternative scenarios: one assuming a halved pace of mortality decline relative to the original forecast and the other assuming a doubled pace of decline (scenarios depicted in Fig. S11).

Table S7 presents the average contributions to changes in cohort life expectancy, complementing the information shown in Table S6. This table provides values across contiguous cohorts, grouped into ten-cohort intervals, and across age groups for Switzerland, using the CPS forecasting method. Panel A reports the average age-specific contributions to changes in cohort life expectancy across cohort groups under the original forecasted pace of mortality decline. Panel B displays the corresponding contributions assuming a mortality decline rate twice as fast as the original forecast, while Panel C presents the contributions under a scenario where the rate of decline is reduced by half. When examining the scenarios in which the pace of mortality decline is either doubled or halved, the largest contributions to changes in cohort life expectancy are concentrated at older ages (60–85+) and among the youngest cohorts (1991–2000). This pattern arises because these cohorts and age groups are subject to longer forecasting horizons; thus, amplifying or reducing the rate of decline has a greater cumulative effect on projected mortality trends, as illustrated in Fig. S10. In contrast, for younger and middle-aged individuals, the forecasted trajectories are shorter, resulting in comparatively smaller changes in cohort life expectancy under these alternative scenarios.

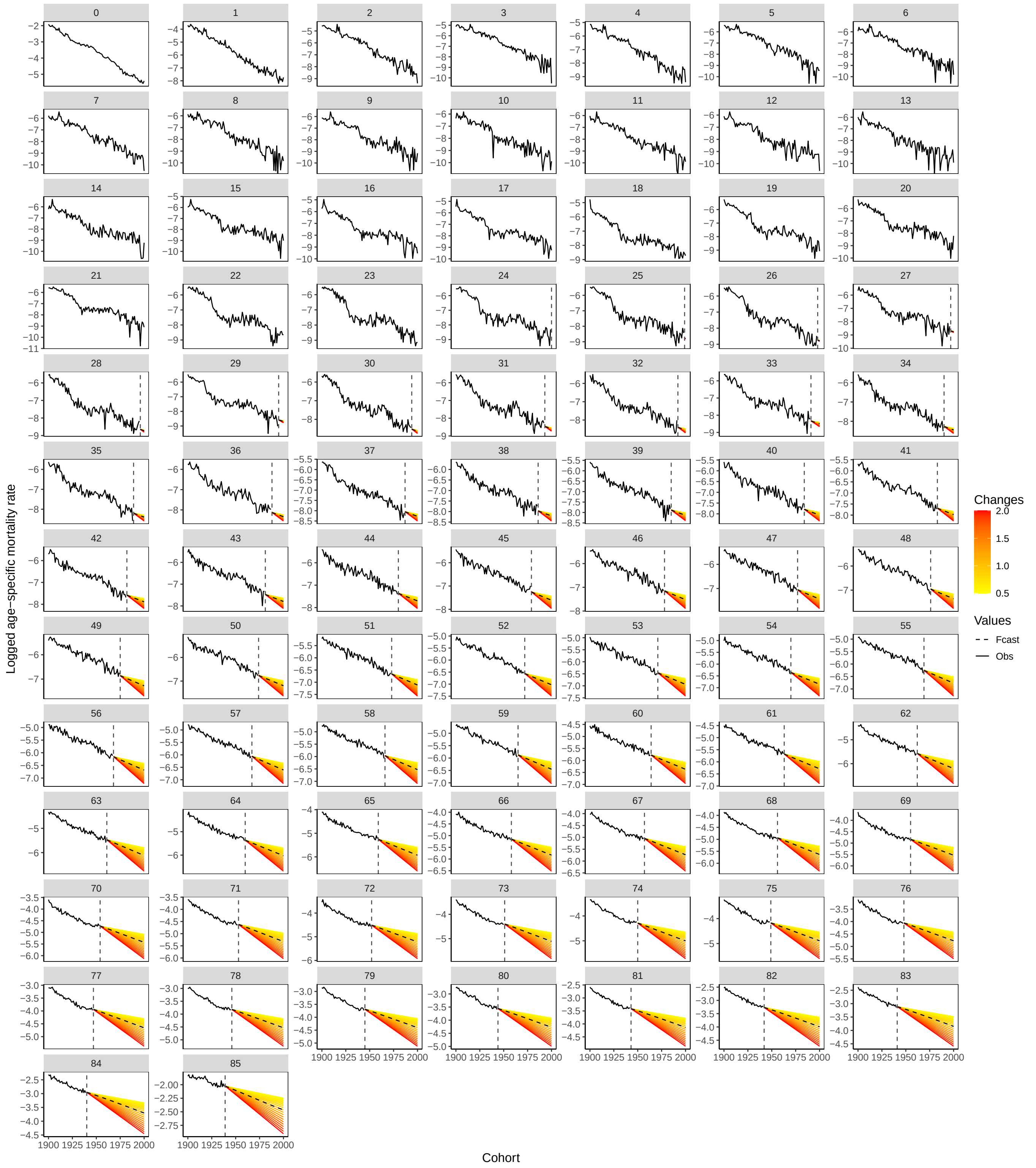

Fig. S10. Future mortality scenarios changing forecasted ROIs in ages 24–85+ in Switzerland using CPS forecasting method

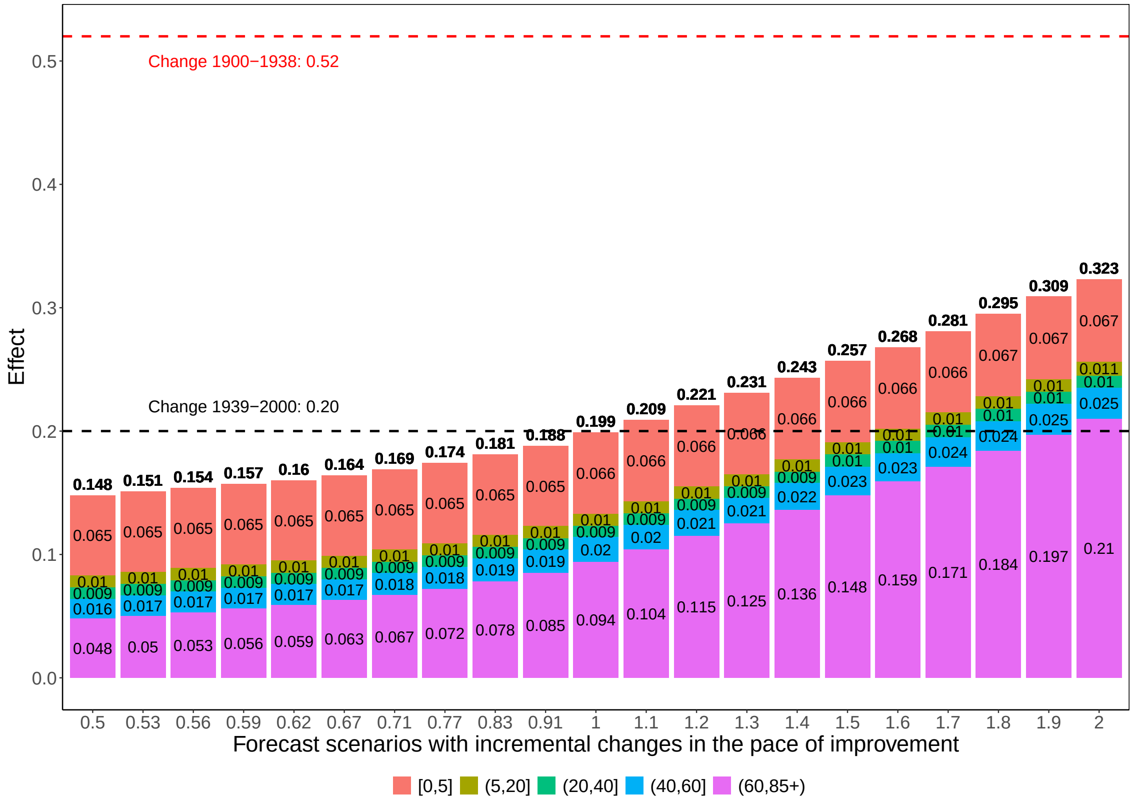

Fig. S11. Decomposition of cohort life expectancy by age groups for cohorts born between 1939 and 2000 in Switzerland, based on forecasted ROIs scenarios changing age-specific mortality rates from ages 24 to 85+ using the CPS forecasting method. The black dashed line represents the average change in cohort life expectancy for cohorts born between 1939 and 2000, while the red dashed line represents the average change for cohorts born between 1900 and 1938.

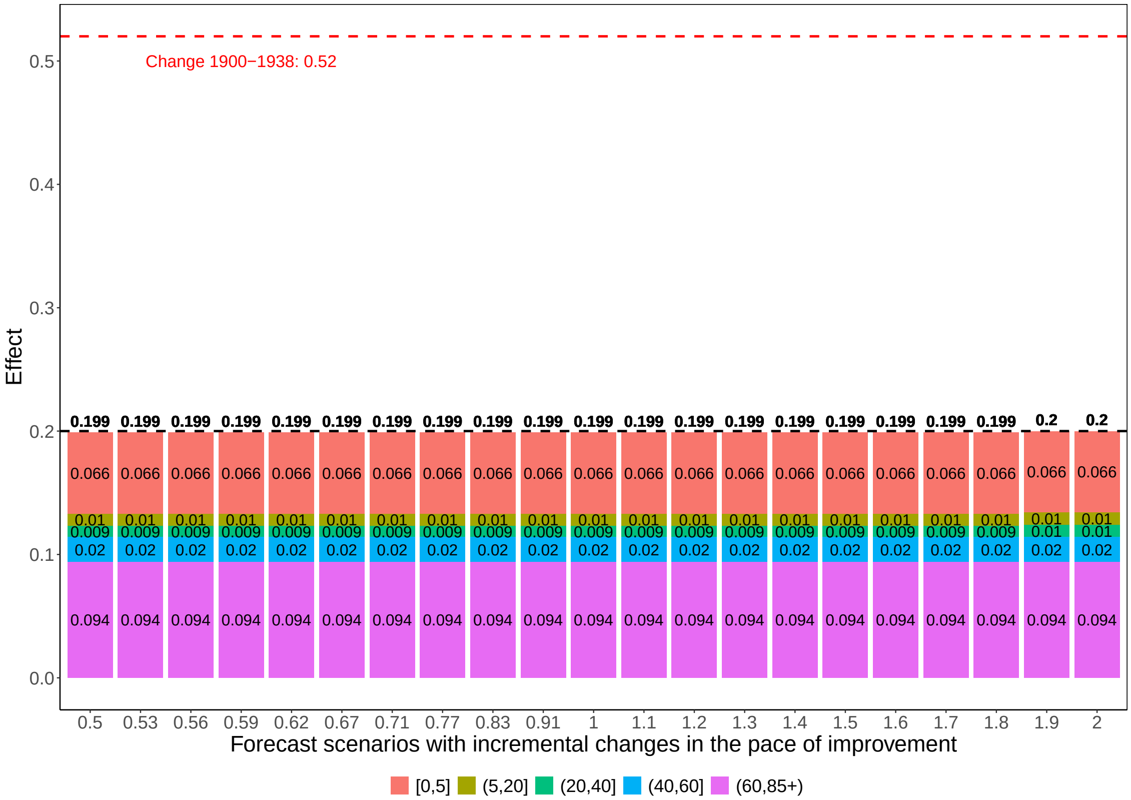

Fig. S12. Decomposition of cohort life expectancy by age groups for cohorts born between 1939 and 2000 in Switzerland, based on forecasted ROIs scenarios changing age-specific mortality rates from ages 24 to 40 using the CPS forecasting method. The black dashed line represents the average change in cohort life expectancy for cohorts born between 1939 and 2000, while the red dashed line represents the average change for cohorts born between 1900 and 1938.

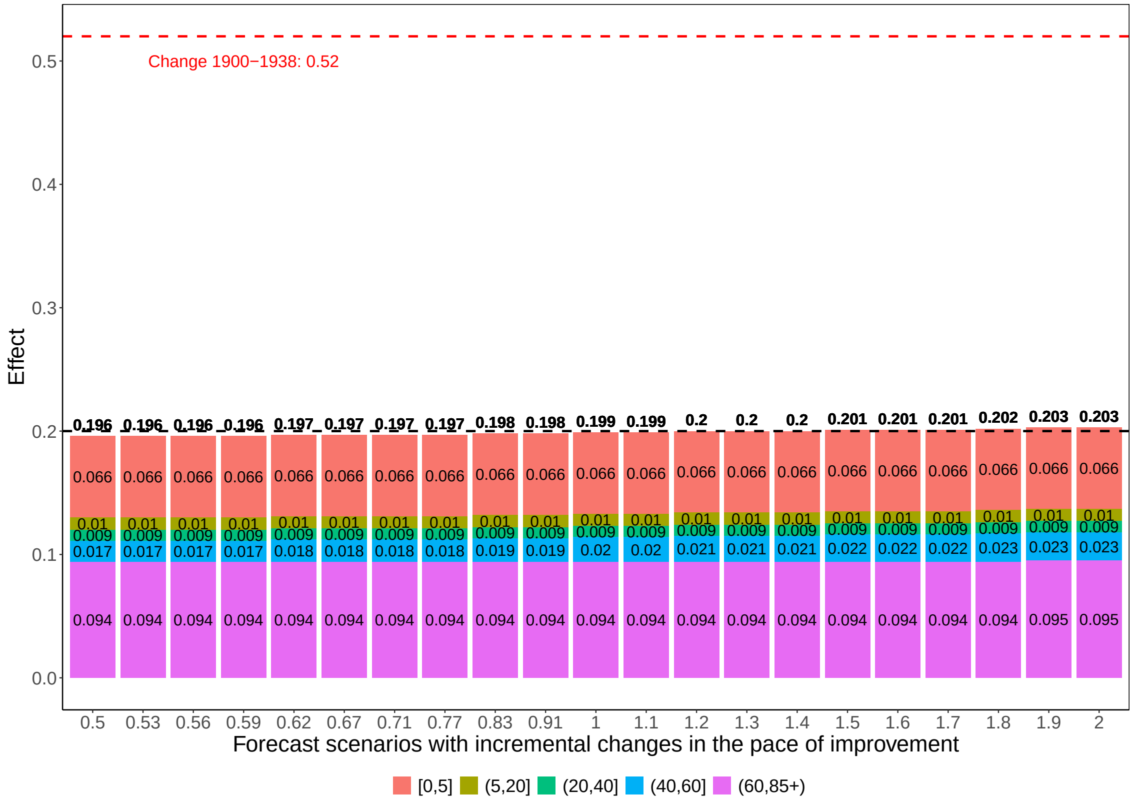

Fig. S13. Decomposition of cohort life expectancy by age groups for cohorts born between 1939 and 2000 in Switzerland, based on forecasted ROIs scenarios changing age-specific mortality rates from ages 41 to 60 using the CPS forecasting method. The black dashed line represents the average change in cohort life expectancy for cohorts born between 1939 and 2000, while the red dashed line represents the average change for cohorts born between 1900 and 1938.

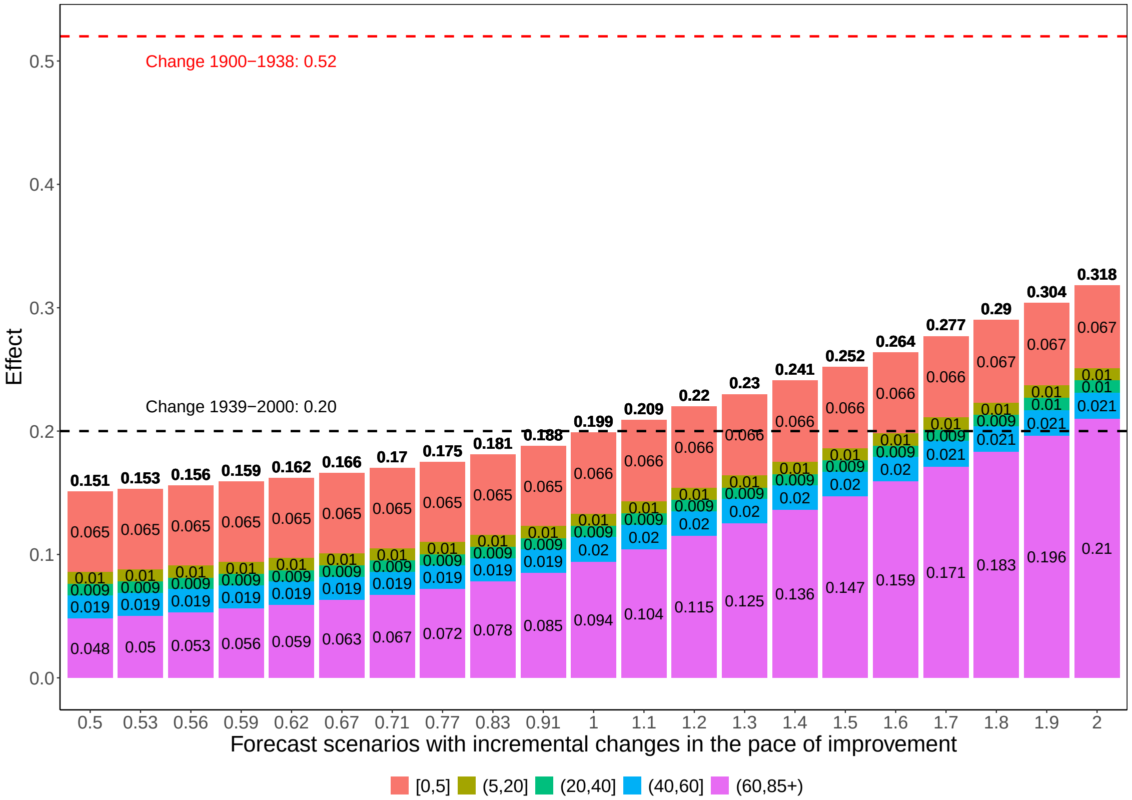

Fig. S14. Decomposition of cohort life expectancy by age groups for cohorts born between 1939 and 2000 in Switzerland, based on forecasted ROIs scenarios changing age-specific mortality rates from ages 61 to 85+ using the CPS forecasting method. The black dashed line represents the average change in cohort life expectancy for cohorts born between 1939 and 2000, while the red dashed line represents the average change for cohorts born between 1900 and 1938.

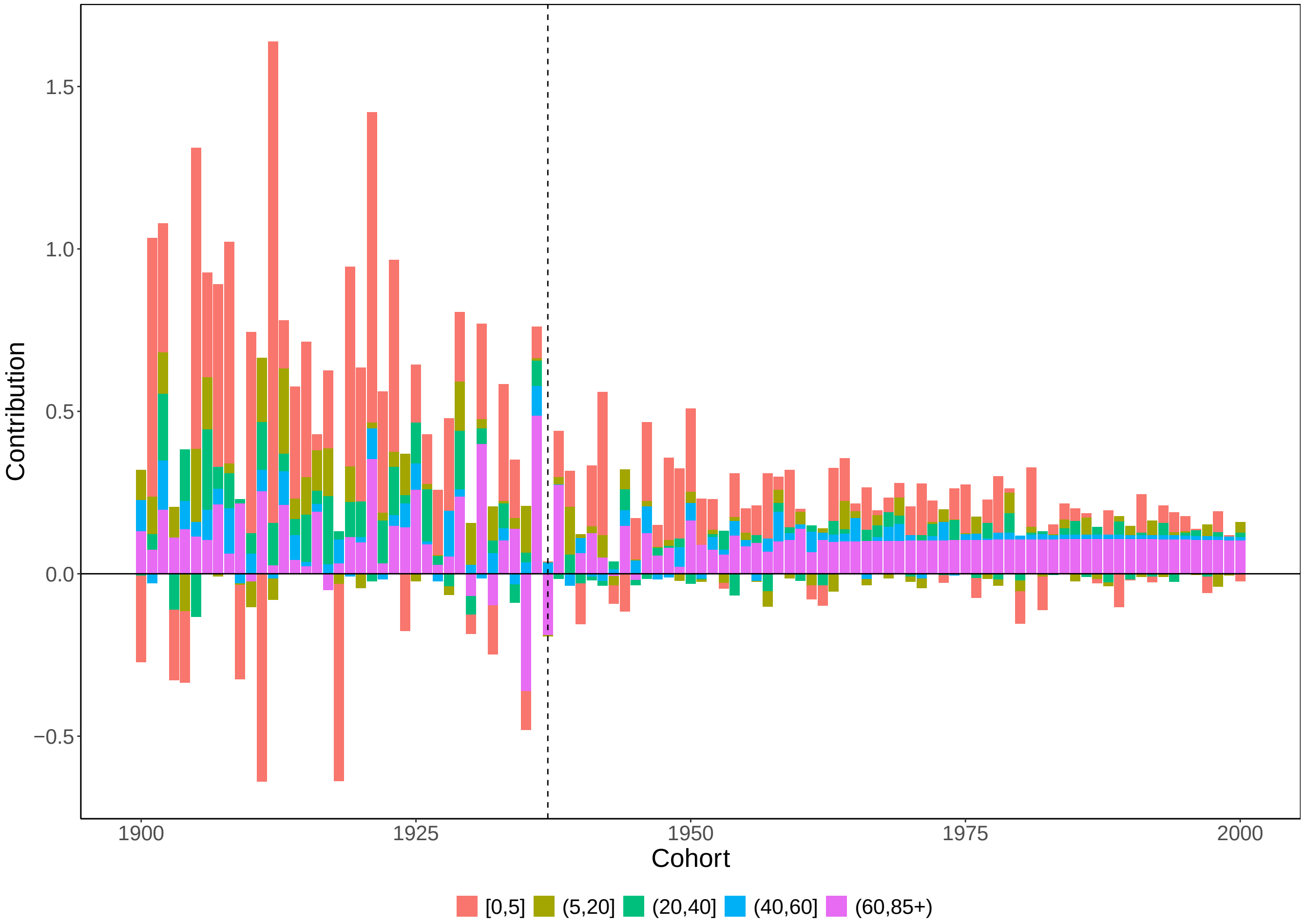

Fig. S15. Contiguous cohort decomposition by age in Switzerland from 1900 to 2000. Before the vertical dashed line (1938), all cohorts are fully observed; after the line, cohorts are completed using the CPS forecasting method.

Table S5. Average contributions of changes in cohort life expectancy by single ages

Age	1900-1938	1939-2000	Difference	Difference (%)
Total	0.517	0.199	0.319	
0	0.167	0.047	0.119	0.374
1	0.041	0.006	0.035	0.110
2	0.012	0.005	0.007	0.023
3	0.007	0.003	0.003	0.011
4	0.006	0.002	0.003	0.011
5	0.006	0.002	0.004	0.013
6	0.004	0.002	0.003	0.009
7	0.002	0.002	0.001	0.002
8	0.002	0.001	0.001	0.004
9	0.002	0.001	0.001	0.003
10	0.003	0.001	0.002	0.006
11	0.002	0.000	0.002	0.006
12	0.003	0.000	0.003	0.009
13	0.003	0.000	0.003	0.009
14	0.003	0.000	0.002	0.006
15	0.004	0.000	0.004	0.011
16	0.004	0.001	0.004	0.012
17	0.004	0.001	0.004	0.012
18	0.005	0.000	0.005	0.016
19	0.011	0.001	0.011	0.034
20	0.005	0.000	0.005	0.016
21	0.005	0.000	0.005	0.015
22	0.004	0.000	0.004	0.013
23	0.004	0.000	0.004	0.012
24	0.004	0.000	0.003	0.010
25	0.004	0.000	0.003	0.010
26	0.004	0.000	0.004	0.012
27	0.004	0.000	0.003	0.010
28	0.003	0.000	0.003	0.009
29	0.004	0.000	0.003	0.010
30	0.004	0.000	0.003	0.010
31	0.003	0.000	0.003	0.008
32	0.004	0.000	0.003	0.010
33	0.003	0.000	0.003	0.010
34	0.003	0.001	0.002	0.007
35	0.003	0.000	0.002	0.007
36	0.003	0.000	0.003	0.009
37	0.002	0.001	0.001	0.004
38	0.002	0.001	0.002	0.005
39	0.001	0.001	0.001	0.002
40	0.002	0.001	0.002	0.005
41	0.002	0.001	0.002	0.006
42	0.002	0.001	0.001	0.004
43	0.002	0.001	0.001	0.005
44	0.002	0.001	0.001	0.003
45	0.002	0.001	0.001	0.004
46	0.002	0.001	0.001	0.004
47	0.002	0.001	0.001	0.004
48	0.002	0.001	0.001	0.003
49	0.002	0.001	0.002	0.005
50	0.002	0.001	0.001	0.004
51	0.002	0.001	0.001	0.004
52	0.002	0.001	0.001	0.003
53	0.002	0.001	0.001	0.003
54	0.002	0.001	0.001	0.002
55	0.003	0.001	0.002	0.005
56	0.002	0.001	0.001	0.002
57	0.002	0.001	0.001	0.003
58	0.002	0.001	0.001	0.003
59	0.002	0.001	0.001	0.003
60	0.003	0.001	0.001	0.005
61	0.003	0.001	0.002	0.005
62	0.003	0.002	0.001	0.003
63	0.004	0.001	0.002	0.007
64	0.003	0.001	0.002	0.006
65	0.003	0.002	0.001	0.004
66	0.004	0.002	0.002	0.007
67	0.004	0.001	0.003	0.009
68	0.004	0.002	0.002	0.007
69	0.004	0.002	0.002	0.006
70	0.004	0.002	0.003	0.008
71	0.004	0.002	0.002	0.007
72	0.005	0.002	0.003	0.010
73	0.004	0.002	0.002	0.008
74	0.005	0.002	0.003	0.009
75	0.005	0.002	0.003	0.008
76	0.004	0.002	0.002	0.007
77	0.004	0.002	0.002	0.006
78	0.005	0.002	0.003	0.008
79	0.004	0.002	0.002	0.006
80	0.004	0.002	0.002	0.006
81	0.004	0.003	0.001	0.005
82	0.004	0.003	0.001	0.003
83	0.004	0.003	0.001	0.004
84	0.003	0.003	0.000	0.000
85+	0.015	0.047	-0.031	-0.098

Average contributions of changes in cohort life expectancy across contiguous cohorts by cohort groups and single ages, for Switzerland. Difference in average contributions and percentage difference. The CPS method is used to forecast cohorts.

Table S6. Average contributions of changes in cohort life expectancy by age groups (selected countries)

Country	Age group	1900-1938	1939-2000	Difference	Difference (%)	Difference (cum %)
**Switzerland**	Total	0.52	0.2 (0.15, 0.32)	0.32 (0.37, 0.19)		
	[0,5]	0.24	0.07 (0.06, 0.07)	0.17 (0.17, 0.17)	0.54	0.54
	(5,20]	0.06	0.01 (0.01, 0.01)	0.05 (0.05, 0.05)	0.16	0.70
	(20,40]	0.07	0.01 (0.01, 0.01)	0.06 (0.06, 0.06)	0.18	0.88
	(40,60]	0.04	0.02 (0.02, 0.03)	0.02 (0.03, 0.02)	0.07	0.95
	(60,85]	0.11	0.09 (0.05, 0.21)	0.02 (0.06, -0.1)	0.05	1.00
**Finland**	Total	0.63	0.24 (0.15, 0.32)	0.38 (0.48, 0.3)		
	[0,5]	0.26	0.12 (0.12, 0.12)	0.14 (0.14, 0.14)	0.37	0.37
	(5,20]	0.11	0.02 (0.01, 0.02)	0.09 (0.09, 0.09)	0.24	0.60
	(20,40]	0.11	0.01 (0.01, 0.01)	0.1 (0.1, 0.1)	0.25	0.86
	(40,60]	0.04	0.02 (0.02, 0.03)	0.02 (0.02, 0.01)	0.06	0.91
	(60,85+)	0.11	0.08 (0.04, 0.17)	0.03 (0.07, -0.06)	0.09	1.00
**France**	Total	0.58	0.25 (0.15, 0.32)	0.33 (0.43, 0.26)		
	[0,5]	0.26	0.11 (0.11, 0.11)	0.16 (0.16, 0.15)	0.47	0.47
	(5,20]	0.07	0.01 (0.01, 0.01)	0.06 (0.06, 0.06)	0.17	0.64
	(20,40]	0.09	0.01 (0.01, 0.01)	0.08 (0.08, 0.08)	0.23	0.87
	(40,60]	0.05	0.02 (0.02, 0.03)	0.03 (0.03, 0.02)	0.09	0.96
	(60,85+)	0.11	0.1 (0.04, 0.22)	0.01 (0.06, -0.11)	0.04	1.00
**Sweden**	Total	0.48	0.19 (0.15, 0.32)	0.29 (0.34, 0.16)		
	[0,5]	0.21	0.06 (0.06, 0.06)	0.15 (0.15, 0.15)	0.51	0.51
	(5,20]	0.08	0.01 (0.01, 0.01)	0.07 (0.07, 0.07)	0.25	0.76
	(20,40]	0.07	0.01 (0.01, 0.01)	0.06 (0.06, 0.06)	0.22	0.98
	(40,60]	0.03	0.02 (0.02, 0.03)	0.01 (0.02, 0.01)	0.04	1.02
	(60,85+)	0.09	0.1 (0.05, 0.2)	-0.01 (0.04, -0.11)	-0.02	1.00

Average contributions of changes in cohort life expectancy across contiguous cohorts by cohort groups and age groups. Difference in average contributions, percentage difference, and cumulative percentage difference. The CPS method is used to forecast cohorts. Values in parentheses represent contributions (column 3) and differences (column 4) under two alternative scenarios: one assuming a halved pace of mortality decline relative to the original forecast and the other assuming a doubled pace of decline.

Table S7. Average contributions of changes in cohort life expectancy across contiguous cohorts by cohort groups under different forecast paces.

Age group	1900–1910	1911–1920	1921–1930	1931–1938	1939–1950	1951–1960	1961–1970	1971–1980	1981–1990	1991–2000
**Panel A. Original pace of forecast**										
Total	0.66	0.57	0.56	0.29	0.26	0.21	0.20	0.19	0.15	0.16
[0,5]	0.36	0.25	0.27	0.10	0.13	0.09	0.05	0.05	0.02	0.03
(5,20]	0.05	0.09	0.05	0.04	0.03	0.00	0.01	0.01	0.01	0.01
(20,40]	0.07	0.10	0.07	0.02	0.01	-0.00	0.02	0.02	0.01	0.01
(40,60]	0.06	0.04	0.04	0.03	0.02	0.03	0.03	0.01	0.01	0.01
(60,85]	0.12	0.09	0.13	0.09	0.07	0.09	0.10	0.10	0.10	0.11
**Panel B. Double pace of forecast**										
Total	0.66	0.57	0.56	0.29	0.31	0.31	0.33	0.34	0.32	0.34
[0,5]	0.36	0.25	0.27	0.10	0.13	0.10	0.05	0.06	0.02	0.03
(5,20]	0.05	0.09	0.05	0.04	0.03	0.00	0.01	0.01	0.01	0.01
(20,40]	0.07	0.10	0.07	0.02	0.01	-0.00	0.02	0.02	0.01	0.01
(40,60]	0.06	0.04	0.04	0.03	0.02	0.03	0.04	0.03	0.03	0.02
(60,85+)	0.12	0.09	0.13	0.09	0.12	0.18	0.21	0.23	0.25	0.28
**Panel C. Half pace of forecast**										
Total	0.66	0.57	0.56	0.29	0.23	0.17	0.15	0.13	0.09	0.09
[0,5]	0.36	0.25	0.27	0.10	0.13	0.09	0.05	0.05	0.02	0.03
(5,20]	0.05	0.09	0.05	0.04	0.03	0.00	0.01	0.01	0.01	0.01
(20,40]	0.07	0.10	0.07	0.02	0.01	-0.00	0.02	0.02	0.01	0.00
(40,60]	0.06	0.04	0.04	0.03	0.02	0.02	0.03	0.01	0.01	0.01
(60,85+)	0.12	0.09	0.13	0.09	0.05	0.05	0.04	0.05	0.05	0.05

Average contributions of changes in cohort life expectancy across contiguous cohorts by cohort groups and age groups in Switzerland using CPS forecasting method. Panel A presents the average age-specific contributions to changes in cohort life expectancy across cohort groups under the original forecasted pace of mortality decline. Panel B displays the corresponding contributions assuming a doubled rate of decline relative to the original forecast, while Panel C shows the contributions under a scenario with a halved pace of decline.

B. Age-specific mortality rates trends. This section presents the observed and forecasted trends of age groups mortality rates normalized to 1 in 1850 for best-practice (Fig. S16) and median case (Fig. S17). The red dashed line indicates the trends in observed (left) and forecasts (right).

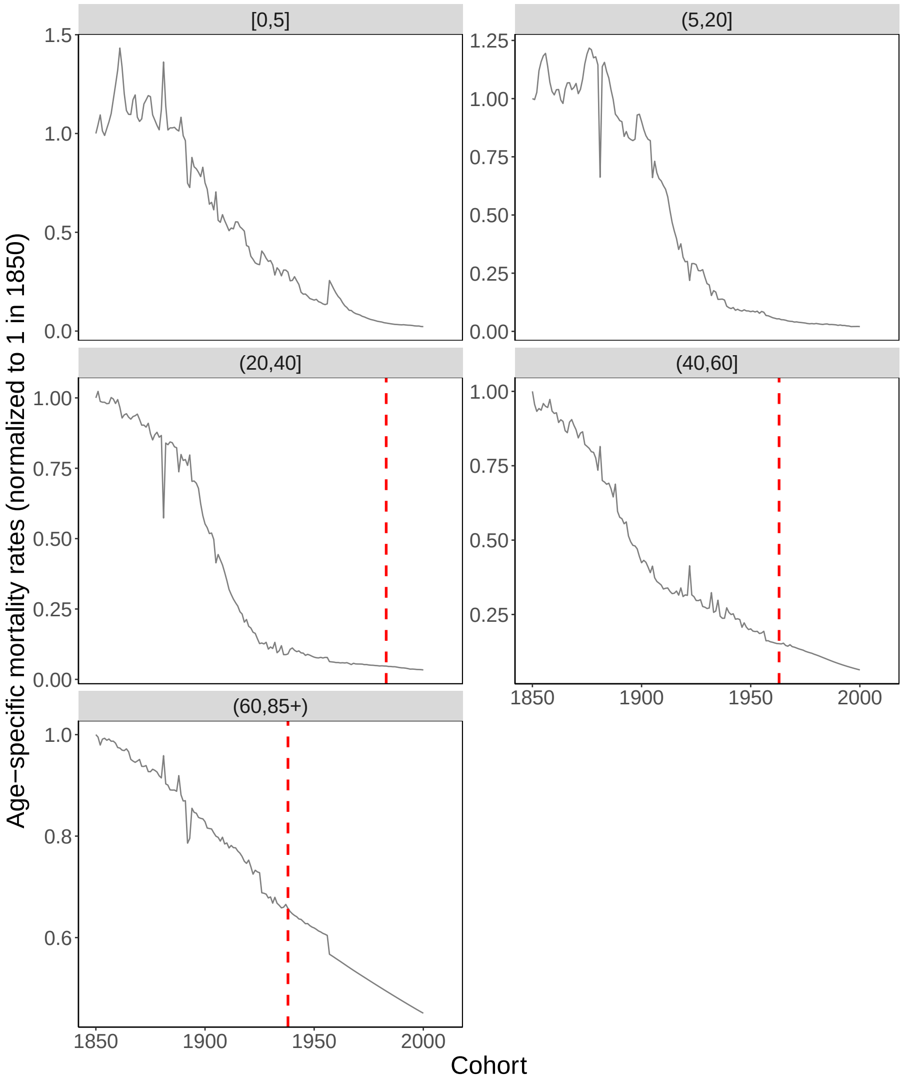

Fig. S16. Trends in best-practice cohort mortality rates, normalized to 1 in 1850 across age groups. Before the solid red line, all values are observed; after the line, values are forecasted using the CPS method.

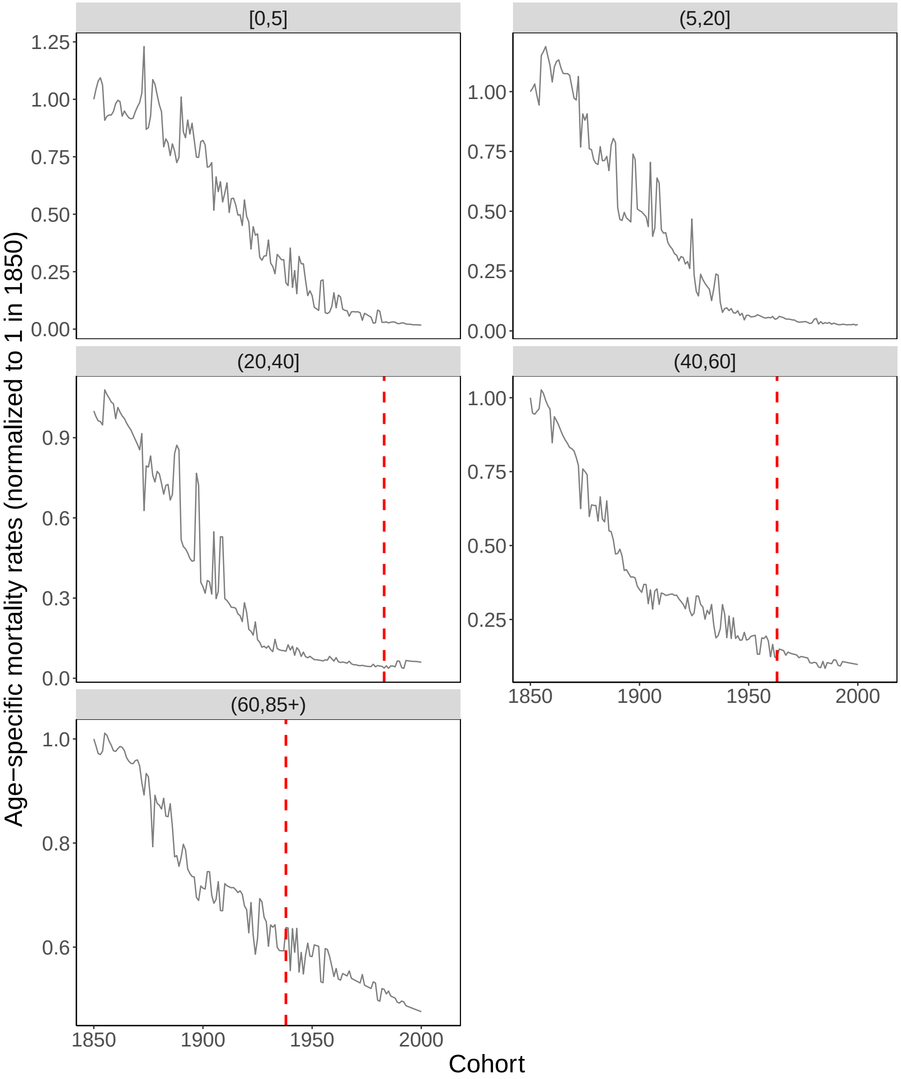

Fig. S17. Trends in median cohort mortality rates, normalized to 1 in 1850 across age groups. Before the solid red line, all values are observed; after the line, values are forecasted using the CPS method.

References

1. MP Bergeron-Boucher, V Canudas-Romo, JE Oeppen, JW Vaupel, Coherent forecasts of mortality with compositional data analysis. *Demogr. Res.* **37**, 527–566 (2017).
2. RD Lee, LR Carter, Modeling and Forecasting U. S. Mortality. *J. Am. Stat. Assoc.* **87**, 659 (1992).
3. CG Camarda, Smooth constrained mortality forecasting. *Demogr. Res.* **41**, 1091–1130 (2019).
4. U Basellini, CG Camarda, The Linear Lee-Carter model to forecast mortality of non-extinct cohorts. *Ext. Abstr. for 2022 Annu. Conf. Popul. Assoc. Am.* (2022).
5. U Basellini, S Kjærgaard, CG Camarda, An age-at-death distribution approach to forecast cohort mortality. *Insur. Math. Econ.* **91**, 129–143 (2020).
6. MC Koissi, AF Shapiro, G Högnäs, Evaluating and extending the lee–carter model for mortality forecasting: Bootstrap confidence interval. *Insur. Math. Econ.* **38**, 1–20 (2006).
